# Effect of a prepartum and postpartum, complex interdisciplinary lifestyle and psychosocial intervention on metabolic and mental health outcomes in women with gestational diabetes mellitus (the MySweetheart trial): randomised, single centred, blinded, controlled trial

**DOI:** 10.1136/bmjmed-2023-000588

**Published:** 2024-02-07

**Authors:** Dan Yedu Quansah, Leah Gilbert, Amar Arhab, Elena Gonzalez-Rodriguez, Didier Hans, Justine Gross, Stefano Lanzi, Bobby Stuijfzand, Alain Lacroix, Antje Horsch, Jardena J Puder

**Affiliations:** 1 Obstetric service, Department Woman-Mother-Child, Lausanne University Hospital, Lausanne, Switzerland; 2 Interdisciplinary Center of Bone Diseases, Bone and Joint Department, Lausanne University Hospital, Lausanne, Switzerland; 3 Service of Endocrinology, Diabetes and Metabolism, Department of Medicine, Lausanne University Hospital, Lausanne, Switzerland; 4 Heart and Vessel Department, Division of Angiology, Lausanne University Hospital, Lausanne, Switzerland; 5 Institute of Higher Education and Research in Healthcare (IUFRS), University of Lausanne, Lausanne, Switzerland; 6 Neonatology service, Department Woman-Mother-Child, Lausanne University Hospital, Lausanne, Switzerland

**Keywords:** Mental health, Metabolic diseases, Public health

## Abstract

**Objective:**

To test the effect of a complex, interdisciplinary, lifestyle and psychosocial intervention on metabolic and mental health outcomes in women with gestational diabetes mellitus during pregnancy and in the post partum.

**Design:**

Single centred, single blinded, randomised, controlled trial (the MySweetheart trial).

**Setting:**

Lausanne University Hospital, Switzerland, from 2 September 2016 to 25 October 2021.

**Participants:**

211 women aged at least 18 years with a diagnosis of gestational diabetes mellitus at 24-32 gestational weeks were randomly assigned (1:1) to the intervention (n=105) or to usual care (n=106).

**Interventions:**

In addition to a comparator based on active guidelines for prepartum and postpartum usual care, the intervention consisted of four individual lifestyle visits during pregnancy and four interdisciplinary visits in the postpartum group, a peer support group workshop in pregnancy and post partum, and a bimonthly lifestyle coach support through telemedicine. The intervention focused on tailored behavioural and psychosocial strategies to improve diet, physical activity, mental health, social support, and adherence to gestational weight gain during pregnancy and weight retention recommendations.

**Main outcome measures:**

Primary outcomes were between-group differences in the decrease in maternal weight and depression symptom scores between baseline and one year post partum. Secondary outcomes included changes in total and central body fat, anxiety, wellbeing, glycaemic parameters (homeostatic model assessment for insulin resistance (known as HOMA-IR) and Matsuda indices), aerobic fitness (maximal oxygen uptake), gestational weight gain, and weight retention. Assessors were blinded to primary and secondary outcomes.

**Results:**

84 (80%) of 105 women in the intervention and 95 (90%) of 106 in the usual care completed the study. There was not enough evidence of a difference in the decrease in weight (mean difference –0.38 kg (95% confidence interval –2.08 to 1.30)) or depression scores (–0.67 (–1.84 to 0.49)). The intervention led to an increase in fat-free mass (0.02 kg (0.01 to 0.03)). The intervention also decreased gestational weight gain since the first gestational diabetes mellitus visit (–1.20 kg (–2.14 to –0.26)) and weekly weight gain throughout the entire pregnancy (–0.14 kg (–0.25 to –0.03)), and led to a higher proportion of women without weight retention at one year post partum (34.1% (28/82) *v* 20.8% (20/96), P=0.034).

**Conclusions:**

Compared with active usual care based on guidelines, there was not enough evidence to conclude that the intervention led to decrease in weight or depression symptoms. However, the intervention decreased gestational weight gain and increased the proportion of women without weight retention.

**Trial registration:**

Clinicaltrials.gov NCT02890693.

**WHAT IS ALREADY KNOWN ON THIS TOPIC**

- Maternal weight retention and depression symptoms increase the risk of diabetes and cardiovascular diseases post partum, particularly in women with gestational diabetes
- Existing lifestyle interventions have shown no or very limited longer term efficacy
- No previous gestational diabetes mellitus intervention has evaluated the effect of a psychosocial and lifestyle interventions on top of current guidelines-based usual care on metabolic and mental health

**WHAT THIS STUDY ADDS**

- No clear evidence was available to show that the 15 month intervention led to a decrease in weight or depression symptoms at one year post partum compared with active guidelines-based usual care
- The intervention led to a significant decrease in gestational weight gain and weight retention and in the need for glucose lowering treatment

**HOW THIS STUDY MIGHT AFFECT RESEARCH, PRACTICE, OR POLICY**

- In women with gestational diabetes mellitus, improvements in metabolic and mental health beyond current guidelines-based care are present after receiving the intervention, but are relatively small

## Introduction

Gestational diabetes mellitus is defined as glucose intolerance that is first diagnosed during pregnancy and does not meet the criteria for overt diabetes.^1^ Due to its high prevalence and impact on maternal and offspring health outcomes and transgenerational transmission of chronic diseases, detection and treatment during and after pregnancy are essential and part of existing guidelines.^1 2^ However, systematic postpartum care remains challenging. About 40% of women are diagnosed with pre-diabetes in the early (six to eight weeks) post partum and gestational diabetes mellitus is associated with long term maternal risks, including a sevenfold higher risk of type 2 diabetes and an increased risk of metabolic syndrome and cardiovascular disease.^3^ Gestational diabetes mellitus is also associated with a higher risk of antenatal and postpartum depression, which increases the risk of weight gain and type 2 diabetes.^4^ Conversely, higher cardiorespiratory fitness and muscular strength are associated with a lower risk of type 2 diabetes and both factors are important predictors of mortality and morbidity.^5^ A global intervention targeting several modifiable risk factors for gestational diabetes mellitus that continues post partum could be key. These include lifestyle factors, such as low physical activity levels, unhealthy diet, sleep disturbances, excessive gestational weight gain, and mental health problems, such as depression .^4^ A meta-review of lifestyle interventions suggests that although they appear to decrease gestational weight gain, current evidence does not show a clear benefit for maternal and infant outcomes in women with overweight or obesity.^6^ Only few lifestyle interventions studies in women with gestational diabetes mellitus exist. Of the seven published trials, two intervened exclusively in pregnancy,^7 8^ three in pregnancy and extended to post partum,^9–11^ and two in post partum only.^12 13^ While some studies reported small differences in gestational weight gain, two short term studies that started in post partum led to a reduction in postpartum weight.^12 13^ One of them included mental health as an outcome and found no difference in depression symptoms between the intervention and usual care groups.^11^ Although recommended,^1 2^ none of these studies included any clinical visits in the post partum for the control group and their control group did not follow the current guidelines. Interventions included diet and physical activity, but not sleep counselling, and physical fitness was not evaluated. To our knowledge, no specific psychosocial interventions were offered. Given that mental health is related to both gestational diabetes mellitus and lifestyle behaviour,^14^ a psychosocial intervention could further improve mental health, lifestyle behaviour, and metabolic health. Outside of the context of gestational diabetes mellitus, few studies have investigated the effect of multidimensional prenatal and perinatal interventions on metabolic outcomes.^15^

Considering the multifactorial origins of gestational diabetes mellitus and the associated risks for the mother, we developed a complex interdisciplinary approach starting in pregnancy and continuing in the post partum. Complex interventions are characterised by several interacting components, several outcomes, a high degree of flexibility, and the possibility of tailoring the intervention. The Health Action Process Approach was chosen as the theoretical framework for this behaviour change intervention.

The aim of this trial was to test the effect of a prenatal and postpartum, complex, interdisciplinary, lifestyle and psychosocial, continuous intervention on metabolic and mental health outcomes in women with gestational diabetes mellitus up to one year post partum. A guidelines-based usual care group during and after pregnancy was also chosen to clarify if a complex intervention beyond current guidelines would lead to additional improvements in the chosen outcomes.

## Methods

### Trial design

The MySweetheart trial was a single blind, randomised, controlled trial that tested the effect of a multidimensional, interdisciplinary, lifestyle and psychosocial intervention during prepartum and post partum on metabolic and mental health outcomes in women with gestational diabetes mellitus compared with treatment as usual. The study protocol was approved by the Human Research Ethics Committee of the Canton de Vaud (study number 2016-00745). More details regarding the study have been published in the study protocol^16^ and the reporting of this study follows the CONSORT statement.

### Participants

Eligible participants included pregnant women diagnosed with gestational diabetes mellitus according to the International Association of Diabetic Pregnancy Study Groups criteria.^17^ Women with gestational diabetes who were least 18 years, between 24-32 weeks' gestational age, and understood French or English were included. We excluded women on strict bed rest, with pre-existing diabetes, or if they had a current severe mental health disorder. Women were recruited at the diabetes in pregnancy clinic of the Lausanne University Hospital (CHUV) or were referred from antenatal care clinics or obstetricians in private practices, after a diagnosis of gestational diabetes mellitus. Data collection and outcomes were measured at the diabetes in pregnancy clinic of the Lausanne University Hospital. All participants signed an informed consent. During the covid-19 lockdown, we suspended recruitment, testing, and follow-up for three months (until 26 May 2020), and partially for an additional two months due to the extension of restriction guidelines. To avoid unforeseen dropouts linked to the second covid-19 wave, we recruited 11 more participants.

### Usual care group

All women were followed-up according to the current gestational diabetes mellitus guidelines of the American Diabetes Association and the Endocrine Society,^1 18^ and according to the National Institute for Care and health Excellence (NICE) guidelines regarding mental health.^2^ Women were seen at 24-32 weeks' gestational age either by a physician, or a diabetes specialist nurse, and followed up until birth.^16^ They received information about gestational diabetes mellitus, adapted recommendations regarding lifestyle changes and gestational weight gain based on the 2009 recommendations of the Institute of Medicine,^19^ and were taught how to perform self-control of blood glucose four times a day (fasting and two hour postprandial). They also had one appointment with a registered dietician that focused on distribution of carbohydrate intake over several meals and snacks, limiting the intake of free sugars to less than 10%, and increasing fibre intake to up to 30 g per day. Women were advised to reduce sedentary behaviour and engage in physical activity according to the Endocrine Society guidelines. We placed a strong focus on behavioural changes. If depression symptom scores were 13 or higher based on the Edinburgh postnatal depression scale, a referral to the psychiatry liaison service was offered. Treatment with insulin, or rarely with metformin, was introduced when glucose concentrations remained above targets according to national guidelines despite lifestyle changes.^20^ At six to eight weeks and at one year post partum, women underwent a 75 g oral glucose tolerance test followed by a clinical visit. Advice on lifestyle changes were provided based on cardio-metabolic laboratory results. Our clinical treatment-as-usual group was a very active guideline based usual care.

### Intervention group

In addition to the usual care, women in the intervention group followed an evidence based interdisciplinary and personalised lifestyle intervention to further improve their diet and physical activity, mental health, social support, and adherence to gestational weight gain and postpartum weight retention recommendations.^14^ In addition to usual care, the intervention programme consisted of four additional individual visits during pregnancy, and four interdisciplinary visits in the post partum, a peer support group workshop both in pregnancy and post partum, and a lifestyle coach support. Meetings with the lifestyle coach, a trained psychologist, took place bimonthly during pregnancy, and every three weeks during post partum until three months post partum and then once a month until one year post partum, mostly through telemedicine. The four additional visits during pregnancy took place during 24-36 weeks' gestational age, were one to two weeks apart, and consisted of two additional visits with a dietician and two visits with a physiotherapist. The first additional dietary visit focused on limiting total, and particularly, saturated fat intake, and the second focused on mindful eating. The visits with the physiotherapists focused on reducing sedentary behaviour and increasing moderate aerobic and resistance physical activity.

The four interdisciplinary visits in the post partum took place at six to eight weeks’ post partum, four months, seven months, and 10 months post partum. One peer support group workshop took place between 32 and 34 weeks' gestational age and then at four months post partum. The psychosocial wellbeing component of the intervention focused on improving mental health and social support over the entire perinatal period. Mental health was targeted by identifying symptoms of depression by use of an Edinburgh postnatal depression scale questionnaire screening and by offering individual sessions with a clinical psychologist to all women who scored 10 or higher. Following this, the lifestyle coach followed up with the goals set by the clinical psychologist.

The lifestyle coach mainly used the Health Action Process Approach model^16^ and motivational interviewing techniques to augment self-efficacy and to provide social support, the latter also through the peer support group workshops. All visits and coaching interventions and recommendations were tailored to the patients' situation and needs. The coach also interacted with the midwives to encourage breastfeeding support.

### Study procedures

The MySweetheart trial mainly consisted of three main participant visits: the first gestational diabetes mellitus visit (baseline, 24-32 weeks gestational age), six to eight weeks post partum, and one year post partum visits. At all visits, validated self-report questionnaires, mental health, physical activity and fitness measures, body composition, and laboratory variables were assessed only in the first and last visit, except for physical activity and fitness measures, which were only assessed at six to eight weeks post partum. At the six to eight weeks and one year postpartum visits, participants underwent a 75 g oral glucose tolerance test (online supplemental tables). Women who gave additional written consent, had a body composition analysis by dual-x-ray absorptiometry at one year post partum.

10.1136/bmjmed-2023-000588.supp1 Supplementary data

#### Sociodemographic variables

Data for maternal sociodemographic characteristics including age, nationality or ethnic origin, and educational level were collected during the first gestational diabetes mellitus visit. Information about previous history of gestational diabetes mellitus, family history of diabetes, gravida, parity, and social support during pregnancy were extracted from participants' medical charts. We extracted pre-pregnancy weight from participants' medical charts and if missing, was self-reported.

#### Body composition measures

Weight was measured to the nearest 0.1 kg with regularly calibrated electronic scales (Seca) and height with the same tool to the nearest 0.1 cm. We defined gestational weight gain as the difference in weight at the end of pregnancy and before pregnancy, while weight retention at one year post partum was calculated as the difference between weight at one year post partum and pre-pregnancy weight. Excessive gestational weight gain was calculated according to Institute of Medicine's gestational weight gain recommendations based on pre-pregnancy body mass index.^19^ Total fat and fat-free mass were estimated from the reactance and resistance values from bioelectrical impedance analysis measures (Akern BIA 101) using the Kyle equation.^21^ Total and regional fat mass were measured by dual x-ray absorptiometry at one year post partum.

#### Metabolic measures

Fasting glucose, insulin, and HbA_1c_ concentrations were measured at 24-32 weeks gestational age. At six to eight weeks and one year post partum, a 75 g oral glucose tolerance test was performed with glucose and insulin sampling at 30 min intervals up to two hours to assess glucose tolerance and insulin sensitivity using HOMA-IR and Matsuda indices. At one year post partum, pre-diabetes (fasting plasma glucose of 5.6-6.9 mmol/L, and/or HbA_1c_ of 5.7-6.4%, and/or two hour plasma glucose of 7.8-11.0 mmol/L) and diabetes (fasting plasma glucose of ≥7.0 mmol/L, two hour glucose of ≥11.1 mmol/L, and/or HbA_1c_ of ≥6.5%) were defined according to the American Diabetes Association criteria.^1^

#### Aerobic and muscular fitness measures

The Chester Step test, a multistage submaximal exercise test, was used to assess aerobic fitness (maximal oxygen uptake (VO_2_max)).^22^ Muscular fitness was assessed with a hand grip strength dynamometer (Jamar). Participants sat in a chair and squeezed the dynamometer as tightly as possible. Three measures were taken for each hand and the highest value of each hand was used for analysis.

#### Mental health measures

The Edinburgh postnatal depression scale was used to measure symptoms of depression in the preceding seven days.^23^ The scale has been validated in pregnant women, also in a French population, and good psychometric properties have been reported.^24^ Maternal wellbeing was assessed with the WHO-five wellbeing index (WHO-5 index), a validated five item self-report questionnaire assessed on a Likert scale ranging from 0 (at no time) to 5 (all of the time). The total score of the five items is then multiplied by four to obtain a final score. Possible scores range from 0 to 100, and higher scores reflect higher wellbeing status. The hospital anxiety and depression scale (HADS) anxiety subscale was used to measure symptoms of anxiety in the preceding seven days.^25^ The seven items of this questionnaire are scored on a four point scale, total score ranged from 0 to 21.

### Primary and secondary outcomes

The primary outcomes were differences in weight between inclusion (24-32 weeks gestational age) and one year post partum and an attenuation in maternal symptoms of depression during the same period between the intervention and the usual care. Assessors were masked to the primary and secondary outcomes except for physical fitness measures. Secondary maternal outcomes included body composition (ie, bioelectrical impedance analysis and dual-energy x-ray absorptiometry), cardio-metabolic laboratory (ie, blood glucose, lipids, and incidence of pre-diabetes or metabolic syndrome at one year post partum), physical fitness measures, and additional mental health variables (HADS) and wellbeing (WHO-5 index)).

### Sample size and randomisation

We estimated the sample size based on the expected differences in primary outcomes (maternal weight and depression) between the usual care and intervention. Regarding weight, we assumed a weight reduction of 8.4 kg (standard deviation 5.5) between enrolment at 24-32 weeks' gestational age and one year post partum in the usual care group compared with 10.9 kg (5.5) in the intervention group. The required sample size for each study group to reach a statistically significant difference with a power of 80% and an alpha level set at 0.05 (two sided) was 76 women. This estimated sample size was also sufficient to observe a mean of a 0.2 (standard deviation 4.3) reduction in depression symptom score between enrolment and one year post partum in the usual care and 2.2 (4.4) in the intervention group. We also assumed a maximum attrition rate of 30%. Thus, we needed to include 100 women in each group to provide adequate power. An allocation ratio of randomisation was 1:1 using the block randomisation method (blocks of four) after stratification. A computer-generated list of random blocks was used to allocate participants into study groups (intervention or usual care). The allocation sequence was concealed in sequentially numbered, opaque, sealed envelopes.

### Statistical analysis

All statistical analyses were done with Stata/SE 15.1. Sociodemographic and medical characteristics were presented as either means (standard deviation)or in absolute numbers (percentages). Primary outcome variables including the decrease in weight and attenuation in maternal symptoms of depression between baseline and one year post partum were normally distributed. We used multiple linear regressions to assess effect estimates of the decrease in weight and attenuation in maternal symptoms of depression between baseline and one year post partum and the most important secondary outcomes and adjusted for the baseline values. We did not correct for multiple testing due to the multifactorial nature of the intervention.

We also compared differences in further secondary outcomes between the intervention and usual care using Fischer's exact test for categorical variables, which were the proportion of women meeting the Institute of Medicine's recommendations, weight retention (yes/no) at one year post partum, and symptoms of depression categories (minimal, moderate, or elevated) at one year post partum. We used multiple linear regression to estimate the differences in outcomes for continuous variables: gestational weight gain after the first gestational diabetes mellitus visit, total gestational weight gain, and rate of weight gain per week during the entire pregnancy. In all analysis, we adjusted for gestational age at randomisation and for the timing of the one year visit. In a second analysis, we further adjusted for maternal age, migrant status (born in Switzerland *v* elsewhere), and educational level, if any of these variables were related (P<0.20) to the outcome. We calculated effect estimates based on the differences in outcome between the one year post partum and baseline results except for measures that were not assessed at baseline (fat mass (dual x-ray absorptiometry), visceral adipose tissue (dual x-ray absorptiometry), two hour glucose values after oral glucose tolerance test, and Matsuda index). No imputations for missing data were done. In addition to the intention-to-treat analyses, we also performed per-protocol analyses for the primary and secondary outcomes.

In an ancillary subgroup analysis, we stratified the analysis with high risk status (high risk denotes women who were overweight or obese (body mass index of ≥25 kg/m^2^) before pregnancy and had moderate to elevated depression score (Edinburgh postnatal depression scale ≥10) at baseline (yes/no)) and compared the primary outcomes between the intervention and usual care and also tested for an interaction effect. We also did two exploratory analyses using multiple linear regressions to assess the effectiveness of the intervention on the primary outcomes and most important secondary outcomes between the baseline and six to eight weeks postpartum visits as well as between the six to eight weeks and one year postpartum visits (online supplemental tables). All statistical significance was two sided and accepted at P<0.05.

### Patient and public involvement

The components of the intervention and the decision for the choice of the primary and key secondary outcomes were informed with the input of the clinical healthcare team and three informal focus group sessions with patients (pregnant women with gestational diabetes mellitus and women post partum). This was done to better understand the needs of these women and how they would like to be supported. Thereby, the options to have support sessions with a lifestyle coach (ideally by phone), interdisciplinary visits, diabetes educators, and dietitians, and peer support workshops were put forward as being most important factors by the patients. These parts of the intervention were therefore also used for the per-protocol analyses (minimal protocol requirement: at least two phone sessions with the lifestyle coach in the prepartum and three phone sessions in the post partum, attendance of at least one out of four postpartum interdisciplinary visits, and at one of two workshops (either pregnancy or post partum)). Interested participants were informed about the study results.

## Results

**Figure 1:**
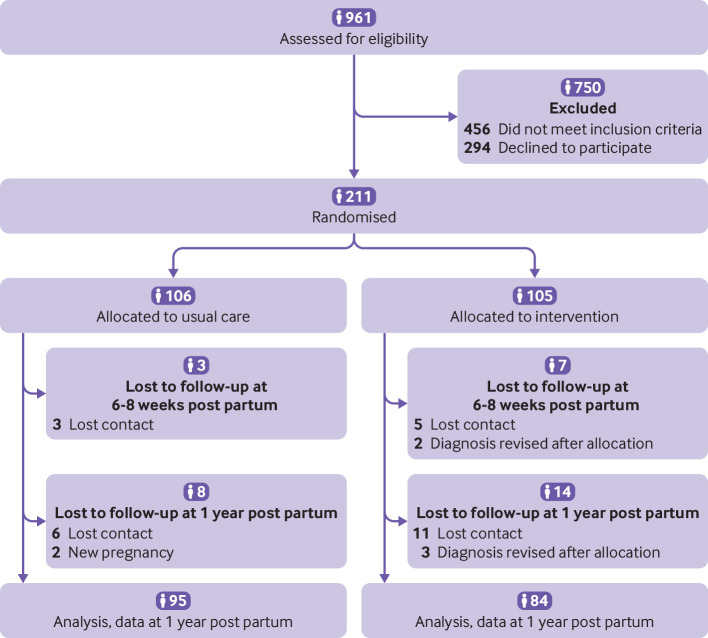
Flow chart of study participants

Of the 961 women with gestational diabetes mellitus, 211 participants met inclusion criteria and were included (105 were randomly assigned to the intervention and 106 to usual care). Of these, 179 (85%) women completed the study (figure 1): 84 (80%) of 105 in the intervention group and 95 (90%) of 106 in the usual care group. All 179 women had complete and valid data for both primary outcomes. Overall, 32 (15%) of 211 women dropped out of the study. Of these, 24 (75%) of 32, dropped out because of time constraints or because they were either unreachable, moved from the city, or were admitted to hospital. Six (19%) of 32 had new pregnancies (before one year post partum) and for two (6%) of 32, the diagnosis of gestational diabetes mellitus had to be revised and women were not eligible anymore. Regarding the timepoints, six women dropped out after the baseline visit, four before the first postpartum visit (six to eight weeks post partum), eight immediately after the first postpartum visit, and 14 between four months and one year post partum. Baseline characteristics did not differ between women who did and did not drop out (data not shown). The intervention started at 29.0 (plus or minus 2.3) weeks’ gestational age and ended at one year post partum. The first patient recruitment started on 2 September 2016 and the last patient's one year follow-up visit was 25 October 2021. Baseline sociodemographic and clinical characteristics were similar between both groups. There were no significant differences in age, gestational age at randomisation, body mass index, history of previous gestational diabetes mellitus, family history of diabetes, and parity (table 1). Regarding the need for glucose lowering medication during pregnancy, women in the intervention group had a lower need of insulin treatment during pregnancy (32% (34/105) *v* 42% (45/106), P=0.040) but not of metformin (9% (9/105) *v* 2% (2/106)) than the usual care group.

**Table 1:** Baseline maternal sociodemographic and clinical characteristics between intervention and usual care groups. Data are numerator (%), unless otherwise specified

Variable	All (n=211)	At randomisation		Included in analysis*	
		Intervention (n=105)	Usual care (n=106)	Intervention (n=84)	Usual care (n=95)
Age (year), mean (SD)	33.63 (4.9)	34.48 (5.1)	32.79 (4.6)	34.68 (5.1)	32.60 (4.6)
Gestational age at the first GDM visit (weeks) mean (SD)	29.0 (2.3)	28.88 (2.3)	29.10 (2.4)	28.83 (2.1)	29.13 (2.4)
Pre-pregnancy weight (kg) mean (SD)	69.76 (15.5)	69.87 (14.8)	69.66 (16.3)	69.15 (14.3)	69.36 (16.5)
Pre-pregnancy BMI (kg/m^2^) mean (SD	25.89 (5.4)	25.92 (5.4)	25.86 (5.5)	25.49 (5.2)	25.84 (5.5)
BMI at the first GDM visit (kg/m^2^) mean (SD)	29.68 (5.0)	29.53 (4.8)	29.82 (5.2)	29.10 (4.9)	29.77 (5.3)
GWG up to baseline visit (kg) mean (SD)	10.23 (6.12)	9.82 (6.7)	10.64 (5.38)	9.79 (6.28)	10.47 (5.2)
Nationality or ethnic group:					
Switzerland	62 (29)	32 (31)	30 (28)	26 (31)	29 (31)
Rest of Europe and North America	83 (39)	39 (37)	44 (42)	31 (37)	38 (40)
Asia and Oceania	14 (7)	7 (7)	7 (7)	6 (7)	7 (7)
Africa	23 (11)	9 (9)	14 (13)	7 (8)	12 (13)
Latin America	7 (3)	5 (5)	2 (2)	5 (6)	2 (2)
Others	22 (10)	13 (12)	9 (9)	9 (11)	7 (7)
Education level†:					
Compulsory school incomplete‡	2 (1)	1 (1)	1 (1)	1 (2)	1 (1)
Compulsory school achieved	23 (13)	12 (15)	11 (12)	11 (17)	10 (12)
High school	19 (11)	9 (11)	10 (11)	9 (14)	7 (8)
General and vocational education	42 (24)	19 (23)	23 (24)	12 (19)	21 (24)
University	91 (51)	41 (50)	50 (53)	31 (48)	48 (55)
Parity:					
0	120 (57)	57 (54)	63 (59)	45 (54)	58 (61)
1	57 (27)	31 (30)	26 (25)	26 (31)	24 (25)
2	18 (9)	9 (9)	9 (9)	5 (6)	7 (7)
≥3	16 (8)	8 (8)	8 (8)	8 (10)	6 (6)
Gravida:					
1	88 (42)	42 (40)	46 (43)	35 (42)	42 (44)
2	50 (24)	25 (24)	25 (24)	19 (23)	23 (24)
≥3	73 (35)	38 (36)	35 (33)	30 (36)	30 (32)
GDM in previous pregnancy§:					
Yes	25 (28)	14 (29)	11 (26)	9 (11)	10 (11)
Family history of diabetes¶:					
Yes	136 (65)	63 (60)	73 (69)	43 (52)	68 (70)
Social support during pregnancy:					
Yes	198 (94)	99 (94)	99 (93)	77 (94)	91 (94)

BMI=body mass index; GDM=gestational diabetes mellitus; GWG=gestational weight gain.

*Women that continued the intervention up to one year post partum and were included in the primary outcome analysis.

†23 participants in the intervention and 11 in the control had missing data on education.

‡In Switzerland, compulsory schooling lasts eleven years.

§Only for women who had at least one previous pregnancy.

¶Family history of diabetes consists of those with first degree relationship of the participant (eg, mother, father, brother, sister, daughter, and son). All values are expressed as n, % or mean (and standard deviation).

### Primary and key secondary outcomes

In the primary analysis, there was not enough evidence of a between-group difference in the change in weight up to one year post partum (mean difference −0.38 kg (95% confidence interval −2.08 to 1.30)). This result remained unchanged after adjusting for gestational age at baseline, timing of the one year visit, maternal age, migrant status, and education level. Similarly, the decrease in depression symptoms (−0.67 (−1.84 to 0.49)) did not significantly differ between groups (table 2). The changes in glycaemic parameters, HOMA-IR and Matsuda were not significantly different (all P≥0.301). Although there was not enough evidence of a between-group difference in the change in fat-mass (bioelectrical impedance analysis) (−0.25 kg (−1.54 to 1.04)), the intervention was associated with an increase in fat-free mass, after adjusting for maternal age, migrant status, and education level (0.02 kg, (0.01 to 0.03)). Other metabolic health and physical fitness outcomes including visceral adipose tissue (dual-x-ray absorptiometry), aerobic and muscular fitness were all similar between groups (all P≥0.25). Similarly, the decrease in anxiety and the increase in wellbeing scores did not significantly differ between intervention and usual care groups (all P≥0.21).

**Table 2:** Metabolic and mental health and physical fitness outcomes according to intervention and usual care group at baseline and one year post partum

	First GDM visit at 24-32 weeks gestational age, mean (SD)		One year post partum, mean (SD)		Effect estimate*		
	Intervention (n=105)	Usual care (n=106)	Intervention (n=84)	Usual care (n=95)	Between-group difference in means (95% CI)	P value†	P value‡
Metabolic health:							
Weight (kg)	79.6 (13.3)	80.3 (15.8)	71.9 (14.6)	72.9 (17.4)	−0.38 (−2.08 to 1.30)	0.653	0.712
Fat mass (BIA) (kg)	31.9 (8.9)	32.3 (9.7)	26.2 (9.7)	26.8 (11.3)	−0.25 (−1.54 to 1.04)	0.703	0.706
Fat-free mass (BIA) (kg)	47.4 (5.7)	48.1 (6.9)	45.7 (5.7)	45.4 (6.7)	0.53 (−0.31 to 1.38)	0.217	0.041
Fat mass (DXA) (kg)	—	—	27.9 (10.8)	27.8 (12.2)	−0.22 (−4.6 to 4.2)	0.920	0.851
Visceral adipose tissue (DXA) (kg)	—	—	0.6 (0.4)	0.5 (0.4)	0.08 (–0.09 to 0.25)	0.327	0.493
Fasting glucose (mmol/L)	5.1 (0.5)	4.9 (0.4)	5.3 (0.6)	5.4 (0.6)	−0.09 (–0.26 to 0.08)	0.301	NA
HbA_1c_ (%)	5.2 (0.3)	5.0 (0.3)	5.3 (0.3)	5.2 (0.2)	0.02 (–0.05 to 0.09)	0.581	0.568
Two hour glucose (mmol/L)	—	—	5.9 (1.9)	5.9 (1.4)	0.09 (–0.53 to 0.55)	0.974	NA
HOMA-IR	3.7 (1.9)	3.6 (2.2)	3.0 (1.9)	3.4 (2.6)	−0.09 (–0.60 to 0.41)	0.706	NA
Matsuda	—	—	4.9 (3.0)	4.5 (2.6)	0.47 (–0.58 to 1.53)	0.373	NA
Aerobic and muscular fitness:							
Estimated VO_2_max§ (mLO_2_/kg/min)	37.5 (9.1)	40.3 (9.8)	39.1 (7.8)	39.4 (8.7)	0.09 (–0.24 to 2.68)	0.943	—
Hand grip strength (kg)	56.9 (15.0)	58.2 (13.9)	60.3 (15.3)	59.2 (14.6)	0.37 (–2.92 to 3.68)	0.882	0.891
Mental health:							
Depression symptoms (EPDS)	7.5 (5.0)	7.4 (4.6)	5.5 (4.8)	5.8 (3.6)	−0.67 (–1.84 to 0.49)	0.254	NA
Anxiety symptoms (HADS)	5.9 (4.1)	6.4 (3.8)	5.1 (3.1)	5.9 (3.0)	−0.98 (–2.06 to 0.90)	0.072	NA
Wellbeing (WHO-5)	58.6 (17.7)	56.5 (17.6)	68.4 (16.7)	65.4 (15.7)	3.47 (–1.33 to 8.27)	0.156	0.153

P values are based on linear regression estimates of the change between the first GDM visit and one year post partum. Data are presented as mean (standard deviation). Baseline corresponds to the first GDM visit. BIA=bioelectrical impedance analysis; DXA=dual energy absorptiometry; EPDS=Edinburgh postnatal depression scale score; GDM=gestational diabetes mellitus; HADS=hospital anxiety and depression scale score; HOMA-IR=Homeostatic Model Assessment for Insulin Resistance; NA=not applicable; WHO-5=World Health Organization-five wellbeing index

*Effect estimates are based on the differences in the change between the first GDM visit and one year post partum, except for DXA and Matsuda that were only measured at one year post partum.

†P value for effect estimate adjusted for gestational age at the first GDM visit (baseline), timing of the one year visit, and the baseline value of the outcome.

‡P value for effect estimate adjusted for gestational age at the first GDM visit (baseline), timing of the one year visit, maternal age, migrant status, and education level, baseline value of the outcome (if they were related to the outcome variable).

§VO_2_ max (maximal oxygen consumption), denotes the maximum amount of oxygen that an individual can use during intense or maximal exercise.

Two thirds (69 (66%) of 105) of the women in the intervention group met the criteria for the per protocol analyses. The per protocol analyses did not find any significant between group differences in changes in weight or depression symptoms (table 3). Similarly, secondary outcomes did not differ between groups (table 4).

**Table 3:** Per protocol analysis: metabolic, mental health, and physical fitness outcomes according to intervention and usual care group at baseline and one year post partum

	First GDM visit at 24-32 weeks gestational age		One year post partum		Effect estimate*	
	Intervention (n=69)	Usual care (n=106)	Intervention (n=69)	Usual care (n=95)	Between-group difference in means (95% CI)	P value†
Metabolic health:						
Weight (kg)	78.6 (14.0)	80.3 (15.8)	71.5 (15.2)	72.9 (17.4)	−0.60 (−2.3 to 1.15)	0.497
Fat-mass (BIA) (kg)	31.1 (9.0)	32.2 (9.7)	26.0 (10.0)	26.8 (11.3)	−0.35 (−1.7 to 1.05)	0.618
Fat-free mass (BIA) (kg)	47.5 (6.0)	48.1 (6.9)	45.4 (6.1)	45.4 (6.7)	0.16 (−0.73 to 1.06)	0.716; 0.675‡
Fat mass (DXA) (kg)	—	—	26.9 (10.8)	27.8 (12.2)	−1.16 (−5.9 to 3.6)	0.630
Visceral adipose tissue DXA) (kg)	—	—	0.60 (0.4)	0.54 (0.4)	0.04 (−0.14 to 0.22)	0.649
Fasting glucose (mmol/l)	5.1 (0.55)	4.9 (0.49)	5.3 (0.6)	5.4 (0.6)	−0.12 (0.30 to 0.06)	0.206
HbA_1c_ (%)	5.1 (0.32)	5.0 (0.31)	5.2 (0.3)	5.2 (0.2)	−0.008 (−0.07 to 0.06)	0.806
Two hour glucose (mmol/L)	—	—	5.8 (2.0)	5.9 (1.4)	−0.08 (−0.67 to 0.50)	0.773
HOMA-IR	3.5 (2.0)	3.6 (2.2)	3.0 (1.9)	3.4 (2.6)	−0.12 (−0.67 to 0.42)	0.664
Matsuda	—	—	5.0 (3.0)	4.5 (2.6)	0.52 (−0.61 to 1.65)	0.363
Aerobic and muscular fitness:						
Estimated VO_2_max§ (mL0_2_/kg/min)	36.7 (8.2)	40.2 (9.8)	39.7 (8.2)	39.4 (8.7)	1.13 (−1.66 to 3.93)	0.424
Hand grip strength (kg)	58.5 (15.1)	58.2 (13.9)	61.6 (13.8)	59.2 (14.5)	1.76 (−1.59 to 5.12)	0.300
Mental health:						
Depression symptoms (EPDS)	8.0 (5.0)	7.4 (4.6)	5.3 (4.4)	5.8 (3.6)	−0.90 (−2.09 to 0.28)	0.134
Anxiety symptoms (HADS)	6.7 (4.3)	6.4 (3.8)	5.5 (3.1)	5.9 (3.0)	−0.83 (−2.00 to 0.34)	0.164
Wellbeing (WHO-5)	58.5 (15.4)	56.5 (17.6)	68.8 (16.4)	65.9 (15.7)	4.22 (−0.90 to 9.34)	0.16

P values are based on linear regression estimates of the change between the first GDM visit (baseline) and one year post partum. Data are presented as mean (standard deviations). Baseline corresponds to the first GDM visit. BIA=bioelectrical impedance analysis; DXA=dual energy absorptiometry; EPDS=Edinburgh postnatal depression scale score; GDM=gestational diabetes mellitus; HADS=hospital anxiety and depression scale score; HOMA-IR=homeostatic Model Assessment forInsulin Resistance; NA=not applicable; WHO-5=World Health Organization-five wellbeing index.

*Effect estimates are based on the differences in the change between the first GDM visit and one year post partum, except for DXA and Matsuda that were only measured at one year post partum.

†P value for effect estimate adjusted for gestational age at the first GDM visit (baseline), timing of the one year visit, baseline value of the outcome.

‡P value for effect estimate adjusted for gestational age at the first GDM visit (baseline), timing of the one year visit, maternal age, migrant status and education level, baseline value of the outcome (if they were related to the outcome variable).

§VO_2_ max (maximal oxygen consumption), denotes the maximum amount of oxygen that an individual can use during intense or maximal exercise.

**Table 4:** Per protocol analysis: detailed metabolic and mental health outcomes according to intervention and usual care during pregnancy and at one year post partum

Variable	Intervention (n=69), mean (SD)	Usual care (n=106), mean (SD)	Effect estimate	
			Between-group difference, mean (95% CI)	P value
GWG since first GDM visit (kg)	1.52 (6.4)	3.07 (3.3)	–1.9 (–3.4 to –0.02)	0.002
Total GWG during pregnancy (kg)	10.5 (8.4)	12.5 (6.4)	–1.6 (–3.2 to –0.09)	0.041
Rate of weight gain per week (kg)	0.12 (0.11)	0.31 (0.35)	–0.16 (–0.25 to –0.05)	0.003
IOM recommendation for weight gain since the first GDM visit (kg) (n, %)				
Met recommendation	2 (2.9)	11 (10.4)	—	0.040
Below recommendation	66 (95.7)	91 (85.8)	—	—
Above recommendation	1 (1.4)	4 (3.8)	—	—
IOM's recommendation for total GWG (kg) for the entire pregnancy (n, %):				
Met recommendation	25 (36.2)	27 (25.5)	—	0.144
Below recommendation	23 (33.3)	32 (30.2)	—	—
Above recommendation	21 (30.4)	47 (44.3)	—	—
Weight retention (kg)	2.3 (5.4)	3.9 (6.0)	–1.4 (–3.2 to 0.50)	0.149
Weight retention status at one year post partum (yes/no) (n, %):				
No (≤0 kg)	28 (43.8)	20 (20.8)	—	0.002
Yes (≥0.1 kg)	36 (56.3)	76 (79.2)	—	—
Symptoms of depression at one year post partum (n, %):				
Subclinical symptoms (EPDS<11)	56 (86.2)	84 (86.6)	—	0.556
Probable diagnosis (EPDS≥11)	9 (13.8)	13 (13.4)	—	—

P values for effect estimates are adjusted for age and gestational age at the first GDM visit (baseline), P values for categorical variables are derived from χ^2^ test. CI=confidence interval; EPDS=Edinburgh postnatal depression scale; GDM=gestational diabetes mellitus; GWG=gestational weight gain; IOM=institute of Medicine

Compared with usual care, the intervention had a significantly lower gestational weight gain after the baseline visit (ie, the first gestational diabetes mellitus visit; 1.89 kg (standard deviation 2.9) *v* 3.07 kg (3.3); mean difference −1.20 (95% confidence interval −2.14 to −0.26), table 5). Similarly, the intervention had a lower rate of weekly weight gain throughout pregnancy (0.17 kg (0.32) *v* 0.31 kg (0.35); −0.14 (−0.25 to -0.03)) compared with usual care. However, we observed a non-significant difference of 1.5 kg reduction in total gestational weight gain since the beginning of pregnancy between the intervention and usual care group (mean difference −1.49 (95% confidence interval −3.49 to 0.25)). The proportion of women who met or were below the Institute of Medicine's recommendation for weight gain from the beginning of the study was higher in the intervention compared with usual care group (P=0.018), but this was not the case for the entire pregnancy duration (P=0.25). However, the overall proportion of women meeting the recommendation of a lack of weight retention at one year post partum was higher in the intervention than in the usual care (34% (28/82) *v* 21% (20/96), P=0.034). The proportions of women who breastfed at six to eight weeks (86% (79/92) *v* 83% (84/101) and at one year post partum (38% (18/48) *v* 24% (14/59) were high but not different between groups. No difference was noted in the proportion of women with elevated depression scores at one year post partum in both groups (P=0.42). Results were similar, but more pronounced in the per protocol analyses (table 4). gestational weight gain after the baseline visit and rate of weight gain per week were significantly lower in the intervention group. Similarly, the proportion of women who met or were below the Institute of Medicine's weight gain recommendations and recommendation for lack of weight retention at one year post partum were higher in the intervention group. Additionally, women in the intervention had lower total gestational weight gain during the entire pregnancy.

**Table 5:** Detailed metabolic and mental health outcomes according to intervention and usual care during pregnancy and at one year post partum

Variable	Intervention (n=105)	Usual care (n=106)	Effect estimate	
			Between-group difference, mean (95% CI)	P value
GWG since first GDM visit (kg), mean (SD)	1.89 (2.9)	3.07 (3.3)	−1.20 (−2.14 to −0.26)	0.012
Total GWG during pregnancy (kg), mean (SD)	11.0 (6.2)	12.5 (6.4)	−1.49 (−3.24 to 0.25)	0.093
Rate of weight gain per week (kg), mean (SD)	0.17 (0.32)	0.31 (0.35)	−0.14 (−0.25 to −0.03)	0.008
IOM's recommendation for weight gain since the first GDM visit (kg) (n, %):				
Met recommendation	3 (3)	11 (10)	—	0.018
Below recommendation	100 (95)	91 (86)	—	—
Above recommendation	2 (2)	4 (4)	—	—
IOM's recommendation for total GWG (kg) for the entire pregnancy (n, %):				
Met recommendation	36 (34)	27 (26)	—	0.251
Below recommendation	33 (31)	32 (30)	—	—
Above recommendation	36 (34)	47 (44)	—	—
Glucose lowering treatment in pregnancy (n, %)				
None	62 (59)	59 (56)	—	0.048
Insulin	34 (32)	45 (43)	—	—
Metformin	9 (9)	2 (2)	—	—
Weight retention (kg)	2.9 (5)	3.9 (6)	−0.85 (−2.58 to 0.87)	0.229
Weight retention status at one year post partum (yes/no) (n, %):				
No (≤0 kg)	28 (34)	20 (21)	—	0.034
Yes (≥0.1 kg)	54 (66)	76 (79)	—	—
Breastfeeding at 6-8 weeks post partum, yes (n, %)	79 (86)	84 (83)	—	0.630
Breastfeeding at one year post partum, yes (n, %)	18 (38)	14 (24)	—	0.122
Symptoms of depression at one year post partum (n, %):				
Subclinical symptoms (EPDS <11)	71 (85)	84 (87)	—	0.426
Probable diagnosis (EPDS ≥11)	13 (16)	13 (13)	—	—

P values for effect estimates are adjusted for age and gestational age at the first GDM visit (baseline), P values for categorical variables are derived from χ^2^ test. EPDS=Edinburgh Postnatal Depression scale; GDM=gestational diabetes mellitus; gestational weight gain=gestational weight gain; IOM=Institute of Medicine.

### Exploratory ancillary subgroup analyses

Prespecified subgroup analyses according to prepregnancy body mass index and level of depression symptoms (moderate/elevated depression symptoms) are shown in the online supplemental tables 1 and 2 and they do not substantially differ from the entire population. The intervention decreased gestational weight gain to a similar extent in all body mass index subgroups. Regarding risk categories (normal risk *v* high risk), no significant interactions were recorded between the intervention effect and the risk category for the change in weight (P=0.357) or in depression (P=0.281).

In an exploratory analysis (online supplemental table 3), outcomes (changes between the baseline and at six to eight weeks post partum) did not significantly differ between groups, except for a stronger decrease in HbA_1c_ in the intervention (mean difference −0.52 mmol/L (95% confidence interval −0.69 to −0.36)), (online supplemental table 3). There was no evidence of significant changes in outcomes between six to eight weeks and one year post partum, except for a more pronounced increase in fat-free mass (1.02 kg (0.27 to 1.76)), wellbeing (5.54 (0.07 to 11.02)), and a tendency for a decrease in HbA_1c_ (0.10% (0.004% to 0.21%)) in the intervention group, the latter had similar effect size as the decrease observed beforehand (online supplemental table 4). No adverse events were noted during the study period.

## Discussion

### Principal findings

In this prepartum and postpartum, complex, interdisciplinary, lifestyle and psychosocial intervention in this population of women from multiple ethnic groups with gestational diabetes mellitus, there was not enough evidence to conclude that the intervention decreased weight or depression scores at one year post partum compared with an active lifestyle and mental health guidelines based usual care. However, the intervention led to a decrease in gestational weight gain, a lower rate of weekly weight gain, a higher increase in fat-free mass after adjusting for covariates, and a lower need for insulin treatment. The intervention also increased the proportion of women without weight retention at one year post partum. Results were similar, but more pronounced, in the per-protocol analysis.

### Comparison with other studies

Compared with the previous intervention trials (two exclusively in pregnancy,^7 8^ three in pregnancy and extended to the post partum,^9–11^ and two in the post partum only^12 13^) in women with gestational diabetes mellitus, this is the first multifactorial lifestyle and psychosocial intervention study starting in pregnancy and continuing up to one year post partum.^16^ We used a guidelines based prepartum and postpartum usual care as a control group and included depression symptoms at one year post partum as its co-primary outcome. Our treatment-as-usual group was a more active lifestyle and mental health comparator than those previously reported. The clinical team worked on improving lifestyle behaviours, excessive gestational weight gain, and mental health. This high level of active guidelines based standard care may have reduced the additional benefits of the therapeutic education and the lifestyle and psychosocial components of the intervention, potentially creating a ceiling effect. For example, excess gestational weight gain is usually observed in up to 51% of women with gestational diabetes mellitus.^26^ However, the prevalence of excess gestational weight gain after study inclusion in the usual care group was only 3.8%. Postpartum depression symptoms scores were low in both groups. Our intervention started later (around 29 weeks gestational age) than in other trials^7 8 10^ (<25 weeks gestational age) and thus the window of opportunity during pregnancy to reach such changes was narrow. Based on this potential effect, starting the investigation in the first trimester might have been more beneficial.^27^ We did not define a minimum protocol requirement for the usual care group. However, patients are followed up closely and contacted several times if they would miss an appointment. One requirement for the usual care group was the attendance at the six to eight weeks postpartum care and counselling visit. For this visit, attendance in the usual care group was 97%, which is much higher than in most other settings. Furthermore, the per protocol-analysis concerning the intervention group did not substantially change the results. Breastfeeding rates did not differ between groups, and were high in the intervention group compared with the prevalence of 25% at more than 10 months post partum in Switzerland.^28^ In the subgroup analyses, we dichotomised variables into two groups (to define high risk and low risk), and therefore some information may have been lost compared with a continuous variable analysis.

Data collection for this trial overlapped with the covid-19 lockdown and the potential stress related to the pandemic may have diminished the potential intervention benefits. The similarities in depression scores between groups could be related to the psychosocial advice received by the usual care group. Mean depression scores at one year post partum in both groups were low to moderate and similar to non-gestational diabetes mellitus postpartum populations.^29^ A possible contamination due to improvement of usual care by healthcare providers may have taken place because clinicians participated in the study design and took care of both patients of the usual care and of the intervention group, for whom they regularly exchanged with the lifestyle coach.

The intervention led to a 1.2 kg lower gestational weight gain since study inclusion, a mean of 0.14 kg reduced weekly weight gain in pregnancy and 13% more women did not retain weight at one year post partum. The mean differences in gestational weight gain are of clinical relevance. Some mean differences although not statistically significant, such as for insulin sensitivity (Matsuda), are relevant as well. So far, none of the five existing studies in women with gestational diabetes mellitus that started in pregnancy found any improvements in gestational weight gain, while one improved the proportion of women meeting the Institute of Medicine's gestational weight gain recommendations.^10^ Most (four of six) of existing intervention trials found no differences in weight retention at one year post partum.^7–9 11^ In the two studies that took place only in the post partum,^12 13^ the intervention led to a mean of 3 kg weight loss at one year post partum, although the shorter study duration makes it difficult to tell if this weight difference was maintained. Some other intervention studies found differences at six months, but not at one year post partum. While gestational weight gain is an important predictor for perinatal outcomes,^6^ both gestational weight gain and weight retention are important for long term metabolic health in women with gestational diabetes mellitus.^30^

Mean scores of mental health symptoms were low in both groups, our inclusion of psychosocial and mental health aspects in the multidisciplinary care from late pregnancy onwards should be considered. Mechanistic insight into the most beneficial aspects should be addressed by future research. Future psychosocial and lifestyle interventions should assess interventions starting in the first trimester of pregnancy or even before pregnancy in women of reproductive age with gestational diabetes mellitus risk factors and combine digital technologies with human interventions.

## Conclusions

Although this complex interdisciplinary lifestyle and psychosocial intervention did not lead to differences in the decrease in weight and depression at one year post partum beyond an active guidelines-based usual care, it had a favourable impact on gestational weight gain, need for insulin treatment and weight retention. Even though the active guidelines based standard care may have created a ceiling effect, the intervention could result in additional benefits.

10.1136/bmjmed-2023-000588.supp2 Supplementary data

## Data Availability

Data are available upon reasonable request. Individual participant data collected during the trial (after de-identification) that underlie the publications from MySweetheart research group will be available on reasonable request from the corresponding author (JJP and AH).
