# Supplementary material for: Effect of a prepartum and postpartum, complex interdisciplinary lifestyle and psychosocial intervention on metabolic and mental health outcomes in women with gestational diabetes mellitus (the MySweetheart trial): randomised, single centred, blinded, controlled trial
Source: BMJ Med. 2024 Feb 7;3(1):e000588. doi: 10.1136/bmjmed-2023-000588 (PMC10860000; doi:10.1136/bmjmed-2023-000588)
Supplement: Supplementary data [file bmjmed-2023-000588supp001.pdf]

Supplementary tables/appendix

Table 1: Weight gain according to BMI-categories and recommendations and glucose tolerance according to intervention and usual-care during pregnancy and at 1-year postpartum

Variable	**Intervention (n=105)**	**Usual-care (n=106)**	**Effect estimate mean difference (95% CI)**	**P-value**
	**(n, %)**	**(n, %)**		
GWG since first GDM visit according to pre-pregnancy BMI category (kg) [a]				
Normal	1.95 (3.6)	2.56 (3.2)	0.91 (0.79-1.19)	0.192
Overweight	2.11 (1.6)	3.98 (2.6)	0.70 (0.49-0.99)	**0.047**
Obese	-2.53 (7.9)	-0.97 (13.6)	0.98 (0.93-1.04)	0.651
Recommended weight goal based on pre-pregnancy BMI category (n, %)[b]				
Met recommended goal	18 (17.1)	14 (13.2)	1.3 (0.62-2.89)	0.450
Did not meet the goal	87 (82.9)	92 (86.8)	0.74 (0.35-1.60)	0.545
Glucose tolerance at 1-year pp[c]				
Normal	52 (65.8)	63 (67.7)	0.82 (0.16-4.2)	0.819
Prediabetes	24 (30.4)	27 (29.0)	1.07 (0.55-2.08)	0.826
Diabetes	3 (3.8)	3 (3.2)	1.21 (0.23-6.25)	0.819

GDM denotes gestational diabetes mellitus; IOM denotes Institute of Medicine; GWG denotes gestational weight gain; CI denotes confidence interval, pp denotes postpartum; BMI denotes body mass index

[a]Normal denotes BMI <25kg/m^2, Overweight denotes BMI 25-29.99kg/m^2 and Obese denotes BMI≥30kg/m^2

[b]Met recommended weight goal denotes normal BMI women whose 1-year weight was lower or equal to their pre-pregnancy weight or overweight or obese women who lost at least 5% of pre-pregnancy weight.

[c]Normal denotes fasting glucose <5.6 mmol/l and 2h glucose after OGTT <7.8 mmol/l; Prediabetes denotes fasting glucose <5.6-6.9 mmol/l and 2h glucose after OGTT 7.8-11.0 mmol/l; Diabetes denotes fasting glucose ≥7.0 mmol/l and 2h glucose after OGTT ≥11.0 mmol/l

P-values are adjusted for age and gestational age at the first GDM visit (baseline)

Table 2: Primary mental and metabolic health outcomes at 1-year pp according to high-risk categories stratified by intervention and usual-care groups

	Intervention (n=84) Mean±SD	Usual-care (n=95) Mean±SD	P-value
**Risk categories**			
Normal			
ΔWeight at 1-year pp	-9.1±5.7	-8.7±5.2	0.776
ΔDepression symptoms at 1-year pp	-0.07±4.8	0.23±3.8	0.532
*High risk**			
ΔWeight at 1-year pp	-6.79±6.1	-4.19±4.8	0.233
ΔDepression symptoms at 1-year pp	-8.25±5.6	-5.53±3.29	0.129

BMI denotes body mass index; EPDS denotes Edinburgh Postnatal Depression Scale scores; pp denotes postpartum

*High risk denotes both pre-pregnancy overweight/obese (BMI ≥ 25 Kg/m^2) and moderate/elevated depression score (EPDS ≥10) at baseline

P-value derived from t-test

Data are presented as means±standard deviation.

We investigated if there was an interaction between the allocation group (intervention or control) and the different risk categories (normal risk versus high risk) regarding the effect on our primary outcomes (change in weight and depression at 1-year postpartum) and there were no significant interactions for the change in weight (p=0.357) or in depression (p=0.281).

Table 3: Metabolic and mental health outcomes according to intervention and usual-care group between baseline and at 6-8 weeks postpartum.

	**First GDM visit at 24-32 weeks GA**		**At 6-8 weeks postpartum**		**Effect estimate***	
	**Intervention (n=105)**	**Usual-care (n=106)**	**Intervention (n=98)**	**Usual-care (n=103)**	**Mean difference (95% CI)**	**P-value[a]**
	**Mean±SD**	**Mean±SD**	**Mean±SD**	**Mean±SD**		
Metabolic health						
Weight (kg)	79.6±13.3	80.3±15.8	73.5±13.4	74.5±16.4	-0.64 (-1.65, 0.35)	0.206
Fasting glucose (mmol/l)	5.1±0.5	4.9±0.4	5.1±0.6	5.1±0.7	-0.06 (-0.24, 0.11)	0.482
Fat-mass (BIA)	31.9±8.9	32.3±9.7	28.3±8.9	28.6±10.4	-0.03 (0.87, 0.80)	0.932
Fat-free mass (BIA)	47.4±5.7	48.1±6.9	45.1±5.3	45.9±6.9	-0.40 (-1.10, 0.30)	0.263
HbA1c (%)	5.2±0.3	5.0±0.3	5.2±0.3	5.2±0.4	-0.52 (-0.69, -0.36)	0.021
HOMA-IR	3.7±1.9	3.6±2.2	2.1±1.7	2.2±1.9	-0.13 (-0.59, 0.33)	0.570
Mental health						
Depression symptoms	7.5±5.0	7.4±4.6	5.7±3.6	5.9±4.1	-0.26 (-1.22, 0.70)	0.592
Well-being	58.6±17.7	56.5±17.6	62.6±17.4	63.4±16.5	-1.88 (-6.43, 2.67)	0.416

BIA denotes bioelectrical impedance analysis; CI denotes confidence interval
*Effect estimates are based on the differences in the change between the first GDM visit and 6-8 weeks postpartum
P-values are based on linear regression estimates of the change between the first GDM visit and 6-8 weeks postpartum
[a]P-value for effect estimate are adjusted for gestational age at the first GDM visit (baseline), timing of the 6-8 weeks postpartum visit, baseline value of the outcome
Data are presented as mean±standard deviation.

Table 4: Metabolic and mental health outcomes according to intervention and usual-care group between 6-8 weeks and 1-year postpartum.

	**6-8 weeks postpartum**		**1-year postpartum**		**Effect estimate***	
	**Intervention (n=98)**	**Usual-care (n=103)**	**Intervention (n=84)**	**Usual-care (n=95)**	**Mean difference (95% CI)**	**P-value**
	Mean±SD	Mean±SD	Mean±SD	Mean±SD		
Metabolic health						
Weight (kg)	73.5±13.4	74.5±16.4	71.9±14.6	72.9±17.4	0.24 (-1.19, 1.67)	0.749
Fat-mass (BIA)	28.3±8.9	28.6±10.4	26.2±9.7	26.8±11.3	-0.38 (-1.60, 0.83)	0.537
Fat-free mass (BIA)	45.1±5.3	45.9±6.9	45.7±5.7	45.4±6.7	1.02 (0.27, 1.76)	0.007
Fasting glucose (mmol/l)	5.1±0.6	5.1±0.7	5.3±0.6	5.4±0.6	-0.01 (-0.23, 0.22)	0.925
HbA1c (%)	5.2±0.3	5.2±0.4	5.3±0.3	5.2±0.2	0.10 (-0.004, 0.21)	0.059
2h glucose (mmol/l)	5.4±1.4	5.3±1.3	5.9±1.9	5.9±1.4	0.04 (-0.47, 0.57)	0.853
HOMA-IR	2.1±1.7	2.2±1.9	3.0±1.9	3.4±2.6	0.02 (-0.52, 0.56)	0.939
MATSUDA	7.0±4.1	7.1±3.4	4.9±3.0	4.5±2.6	-0.23 (-1.34, 0.86)	0.668
Weight retention	3.6±6.3	4.7±5.5	2.9±5.2	3.9±6.0	0.22 (-1.24, 1.69)	0.761
Mental health						
Depression symptoms	5.7±3.6	5.9±4.1	5.5±4.8	5.8±3.6	-0.39 (-1.60, 0.81)	0.522
Well-being	62.6±17.4	63.4±16.5	68.4±16.7	65.4±15.7	5.54 (0.07, 11.02)	0.047

BIA denotes bioelectrical impedance analysis
*Effect estimates are based on the differences in the change between the 6-8 weeks visit and 1-year postpartum
P-values are based on linear regression estimates of the change between the 6-8 weeks and 1-year postpartum.
P-value for effect estimate are adjusted for gestational age at the first GDM visit (baseline), timing of the 1-year visit, baseline value of the outcome
Data are presented as mean±standard deviation.

Quansah DY, *et al. BMJMED* 2024; 3:e000588. doi: 10.1136/bmjmed-2023-000588
