# Supplementary material for: Effect of a prepartum and postpartum, complex interdisciplinary lifestyle and psychosocial intervention on metabolic and mental health outcomes in women with gestational diabetes mellitus (the MySweetheart trial): randomised, single centred, blinded, controlled trial
Source: BMJ Med. 2024 Feb 7;3(1):e000588. doi: 10.1136/bmjmed-2023-000588 (PMC10860000; doi:10.1136/bmjmed-2023-000588)
Supplement: Supplementary data [file bmjmed-2023-000588supp002.pdf]

Improving cardio-metabolic and mental health in women with gestational diabetes mellitus and their offspring: *MySweetHeart Study*

Short title: **Gestational diabetes mellitus, cardio-metabolic and mental health**

Study Type:	Randomized controlled trial
Study Categorisation:	Risk Category A
Study Registration:	
Study Identifier:	Not applicable
Principal Investigator:	Prof Jardena Puder, MD, Service EDM, CHUV
Investigational Product:	Not applicable
Protocol Version and Date:	V1 – 04.05.2016

CONFIDENTIAL
The information contained in this document is confidential and the property of the CHUV. The information may not - in full or in part - be transmitted, reproduced, published, or disclosed to others than the applicable Competent Ethics Committee(s) and Regulatory Authority(ies) without prior written authorisation from the sponsor except to the extent necessary to obtain informed consent from those who will participate in the study.

Quansah DY, *et al. BMJMED* 2024; 3:e000588. doi: 10.1136/bmjmed-2023-000588

Signature Page(s)

Study number

Study Title Improving cardio-metabolic and mental health in women with gestational diabetes mellitus and their offspring: *MySweetHeart Study*

The Sponsor-Investigator and trial statistician have approved the protocol version 1 (dated 04.05.2016), and confirm hereby to conduct the study according to the protocol, current version of the World Medical Association Declaration of Helsinki, ICH-GCP guidelines or ISO 14155 norm if applicable and the local legally applicable requirements.

Sponsor-Investigator:
Printed name of Sponsor-Investigator (if Sponsor and PI is not the same person please add an additional signature line for the PI of the study)

Prof Jardena Puder, MD, Service d'endocrinologie, diabétologie et métabolisme, CHUV

Place/Date Signature

Local Principal Investigator at study site*: Not applicable

Table of Contents

STUDY SYNOPSIS **6**
STUDY SUMMARY **10**
ABBREVIATIONS **12**
STUDY SCHEDULE **13**
1. STUDY ADMINISTRATIVE STRUCTURE **15**
1.1. Sponsor, Sponsor-Investigator 15
1.2. Principal Investigator(s) 15
1.3. Statistician ("Biostatistician") 15
1.4. Laboratory 15
1.5. Monitoring institution 15
1.6. Data Safety Monitoring Committee 15
1.7. Any other relevant Committee, Person, Organisation, Institution 15
2. ETHICAL AND REGULATORY ASPECTS **15**
2.1. Study registration 15
2.2. Categorisation of study 15
2.3. Competent Ethics Committee (CEC) 15
2.4. Competent Authorities (CA) 15
2.5. Ethical Conduct of the Study 15
2.6. Declaration of interest 15
2.7. Patient Information and Informed Consent 15
2.8. Early termination of the study 16
2.9. Protocol amendments 16
3. BACKGROUND AND RATIONALE **16**
3.2. Investigational Product (treatment, device) and Indication 20
3.3. Preclinical Evidence 20
3.4. Clinical Evidence to Date 21
3.5. Explanation for choice of comparator (or placebo) 21
3.6. Risks / Benefits 21
3.7. Justification of choice of study population 21
4. STUDY OBJECTIVES **21**
A4.1) Overall Objective 21
A4.2) Primary Objective 21
A4.3) Secondary Objectives 21
A4.4) Safety Objectives 22
B4.1) Overall Objective 22
B4.2) Primary Objective 22
B4.3) Secondary Objectives 22
B4.4) Safety Objectives 22
5. STUDY OUTCOMES **22**
A5.1. Primary Outcomes 22
A5.2. Secondary Outcomes 22
A5.3. Other Outcomes of Interest 22
A5.4. Safety Outcomes 22
B5.1. Primary Outcomes 22
B5.2. Secondary Outcomes 22
B5.3. Other Outcomes of Interest 22
B5.4. Safety Outcomes 22

6. STUDY DESIGN 22
5.1. General study design and justification of design 22
6.2. Methods of minimising bias 23
6.2.1. Randomisation 23
6.2.2. Blinding procedures 23
6.2.3. Other methods of minimising bias 23
6.3. Unblinding Procedures (Code break) 23
7. STUDY POPULATION 23
7.1. Eligibility criteria 23
7.2. Recruitment 24
7.3. Assignment to study groups 24
7.4. Criteria for withdrawal / discontinuation of participants 24
8. STUDY INTERVENTION 24
8.1. Identity of Investigational Products (treatment / medical device) 24
8.1.1. Experimental Intervention (treatment / medical device) 24
8.1.2. Control Intervention (standard/routine/comparator treatment / medical device) 24
8.1.3. Packaging, Labelling and Supply (re-supply) 24
8.1.4. Storage Conditions 24
8.2. Administration of experimental and control interventions 24
8.2.1. Experimental intervention 24
8.2.2. Control Intervention 27
8.3. Dose / Device modifications 27
8.4. Compliance with study intervention 27
8.5. Data Collection and Follow-up for withdrawn participants 28
8.6. Trial specific preventive measures 28
8.7. Concomitant Interventions (treatments) 28
8.8. Study Drug / Medical Device Accountability 28
8.9. Return or Destruction of Study Drug / Medical Device 28
9. STUDY ASSESSMENTS 28
9.1. Study flow chart(s) / table of study procedures and assessments 28
9.2. Assessments of outcomes 28
9.2.1. Assessment of primary outcome 28
9.2.2. Assessment of secondary outcomes 29
9.2.3. Assessment of other outcomes of interest 30
9.2.4. Assessment of safety outcomes 30
9.2.5. Assessments in participants who prematurely stop the study 30
9.3. Procedures at each visit 30
10. SAFETY 31
10.1. Drug studies 31
10.2. Medical Device Category A studies 32
11. STATISTICAL METHODS 32
11.1. Hypothesis 32
11.2. Determination of Sample Size 32
11.3. Statistical criteria of termination of trial 32
11.4. Planned Analyses 33
11.4.1. Datasets to be analysed, analysis populations 33
11.4.2. Primary Analysis 33
11.4.3. Secondary Analyses 33

11.4.4. Interim analyses 34
11.4.5. Safety analysis 34
11.4.6. Deviation(s) from the original statistical plan 34
11.5. Handling of missing data and drop-outs 34
12. QUALITY ASSURANCE AND CONTROL 34
12.1.1. Case Report Forms 34
12.1.2. Specification of source documents 34
12.1.3. Record keeping / archiving 34
12.2. Data management 35
12.2.1. Data Management System 35
12.2.2. Data security, access and back-up 35
12.2.3. Analysis and archiving 35
12.2.4. Electronic and central data validation 35
12.3. Monitoring 35
12.4 Audits and Inspections 35
12.5 Confidentiality, Data Protection 35
12.6. Storage of biological material and related health data 35
13. PUBLICATION AND DISSEMINATION POLICY 36
14. FUNDING AND SUPPORT 36
14.1. Funding 36
14.2. Other Support 36
15. INSURANCE 36
16. REFERENCES 37

STUDY SYNOPSIS

**Sponsor-Investigator**	Prof Jardena Puder, MD, Service d'endocrinologie, CHUV (PI)
**Study Title:**	Improving cardio-metabolic and mental health in women with gestational diabetes mellitus (GDM) and their offspring: *MySweetHeart Study*
**Short Title / Study ID:**	Gestational diabetes mellitus, cardio-metabolic and mental health
**Protocol Version and Date:**	Version 1; 08.04.2016
**Trial registration:**	The study will be registered on clinicaltrials.gov and kofam.ch
**Study category and Rationale**	*MySweetHeart study* is composed of two sub-studies: (A) ***MySweetHeart Trial***: a randomized clinical trial with an intervention entailing minimal risks and burdens (risk category A); (B) ***MySweetHeart Cohort***: an observational cohort study entailing minimal risks and burdens (risk category A).
**Clinical Phase:**	Not applicable
**Background and Rationale:**	Gestational diabetes mellitus (GDM) is a state of glucose intolerance with onset during pregnancy. It is common with prevalence estimates larger than 10% in several populations. GDM carries pre- and perinatal risk for the mother (e.g., pre-eclampsia or preterm delivery) and the infant (e.g. macrosomia or neonatal hypoglycemia) as well as long term risks for the mother (e.g., type 2 diabetes, metabolic syndrome, and cardiovascular disease) and her child (e.g., obesity and type 2 diabetes). Compared to women without GDM, women with GDM are twice as likely to develop perinatal or postpartum depression and approximately one-third of women with recent GDM develop postpartum depression.<br><br>Lifestyle interventions for the treatment of GDM are often limited to physical activity or nutrition, for the mother or the child separately, either only during or only after pregnancy. Their effects are inconsistent. The multifactorial origins of GDM and the tight link between mental and metabolic as well as maternal and child health calls for a multidimensional interdisciplinary approach.<br><br>Furthermore, maternal GDM may be involved in the fetal programming of long-term cardiovascular health. However, evidence is sparse and the effect of GDM on cardiovascular health is not known.<br><br>To address these issues, we will conduct *MySweetHeart Study*, which is composed of two sub-studies **(A)** ***MySweetHeart Trial*** (PI: Prof. J. Puder and Dr A. Horsch) and **(B)** ***MySweetHeart Cohort*** (PI: Prof. N. Sekarski and Dr A. Chiolero; funded by the SNF). Both sub-studies will be conducted in complete coordination.

**Objective(s):**	(A) *MySweetHeart Trial* and (B) *MySweetHeart Cohort* have each specific objectives:<br><br>**A) Overall objective of *MySweetHeart Trial***<br>To test the effect of a multidimensional interdisciplinary lifestyle and psychosocial intervention to improve the cardio-metabolic and mental health of women with GDM and their offspring.<br>**A1) Primary objective of *MySweetHeart Trial***<br>To test the effect of a multidimensional interdisciplinary lifestyle and psychosocial intervention in women with GDM to improve 1) their metabolic (decrease in maternal weight between 24-32 weeks gestational age and the end of the study at 1 yr postpartum) and 2) their mental (decrease in maternal symptoms of depression during the same time period) health.<br>**A2) Secondary objectives of *MySweetHeart Trial***<br>To test the effect of a multidimensional interdisciplinary lifestyle and psychosocial intervention to improve other cardio-metabolic and mental health markers in women with GDM and their offspring.<br><br>**B) Overall objective of *MySweetHeart Cohort***<br>To assess the effect of GDM on offspring cardiovascular health early in life<br>**B1) Primary objective of *MySweetHeart Cohort***<br>To assess the effect of GDM on the surrogate markers of cardiovascular disease (CVD) at birth (left ventricular mass index and subclinical atherosclerosis)<br>**B2) Secondary objectives of *MySweetHeart cohort***<br>To assess the effect of GDM on the cardiovascular structure and function during the fetal period and neonatal adverse cardiovascular risk factors
**Outcome(s):**	**A1) Primary outcomes of *MySweetHeart Trial***<br>Differences between the intervention and the control group in (1) the decrease in maternal weight between 24-32 weeks gestational age and 1 yr postpartum and (2) the decrease in maternal symptoms of the Edinburgh postnatal depression score (EPDS) during the same time period.<br>**A2) Secondary Outcomes *of MySweetHeart Trial***<br>Differences between the intervention and the control group in other maternal secondary outcomes, such as (1) lifestyle behaviours, aerobic fitness and strength, body composition and cardio-metabolic laboratory biomarkers and (2) other mental health indicators during the peri- and postpartum period and offspring secondary outcomes, such as (1) cardio-metabolic laboratory biomarkers at birth, body composition at birth and at 1 yr of age; and (2) mental health indicators at 1 yr of age.<br><br>**B1) Primary outcomes of *MySweetHeart Cohort***<br>Differences in surrogate markers of CVD at birth [left ventricular mass index (LVMI) and subclinical atherosclerosis (carotid intima-media thickness; cIMT)] between offspring of women with GDM and offspring of women without GDM.<br>**B2) Secondary outcomes of *MySweetHeart Cohort***<br>Differences in cardiovascular structure and function during the fetal period (fetal cardiovascular alterations, LVMI, liver volume), and neonatal adverse cardiovascular risk factors between offspring of women with GDM and offspring of women without GDM.
**Study design:**	**A) Study design of *MySweetHeart Trial:***<br>Randomized control trial (RCT) testing the effect of a multidimensional interdisciplinary lifestyle and psychosocial intervention compared with treatment-as-usual in women with GDM and their offspring.<br>**B) Study design of *MySweetHeart Cohort:***<br>Cohort study comparing cardiovascular health between offspring of women with GDM and offspring of women without GDM.
**Inclusion / Exclusion criteria:**	Inclusion criteria: Women aged 18 yrs or older, with or without GDM at 24-32 weeks of gestation, and understanding French or English.<br>Exclusion criteria: Women on strict bed-rest, with pre-existing diabetes or known severe mental disorder

**Measurements and procedures:**	Whenever it is possible, until birth, similar measurements and procedures will be followed for *MySweetHeart Trial* and *MySweetHeart Cohort* (see Table 1 and Table 2 for details).<br><br>**A) *MySweetHeart Trial*:** Patients and their offspring will be followed-up from 24-32 weeks gestational age 1 year postpartum. Once included, patients will be randomly allocated to the usual care (control) group or to the intervention group. The intervention, offered on top of the usual care, consists of individual sessions (face-to-face or telephone contact) with different members of the interdisciplinary team (dietician, physiotherapist, clinical psychologist or coach) and two group sessions. The intervention will consist of dietary and physical activity advice, screening for and treatment of mental health problems, and increase of social support. It will take place during pregnancy and during the first year postpartum. The primary outcomes will be measured at 24-32 wks of gestation and at 1 year postpartum. Secondary and other outcomes will be measured at 30-34 wks of gestation, at birth, at 6-8 wks and 1 year postpartum. Partners will be assessed at study begin and at 1 year postpartum.<br><br>**B) *MySweetHeart Cohort*:** Patients and their offspring will be followed up until a few days after birth. Primary outcomes will be measured a few days after birth. Secondary outcomes will be measured at 30-34 wks of gestation and at birth.
**Study Product / Intervention:**	Not applicable
**Control Intervention (if applicable):**	**A) *MySweetHeart Trial:*** Treatment-as-usual<br>**B) *MySweetHeart Cohort:*** Not applicable
**Number of Participants with Rationale:**	300 women and their offspring in total;<br>**A) *MySweetHeart Trial:*** 100 women with GDM and their offspring in the control group; 100 women with GDM and their offspring in the intervention group.<br>**B) *MySweetHeart Cohort:*** 100 women with GDM and their offspring; 100 women without GDM and their offspring.<br>The 100 women with GDM in the control group of *MySweetHeart Trial* and the 100 women with GDM of *MySweetHeart Cohort* are the same women.
**Study Duration:**	4 years
**Study Schedule:**	06.2016 of First-Participant-In (planned)<br>06.2020 of Last-Participant-Out (planned)
**Investigator(s):**	**A) *MySweetHeart Trial:***<br>Prof. Jardena Puder, MD, Service d'endocrinologie, CHUV<br>Dr Antje Horsch, PhD, Service d'endocrinologie, CHUV<br>**B) *MySweetHeart Cohort:***<br>Prof. Nicole Sekarski, MD, Unité de cardiologie pédiatrique, CHUV<br>PD & MER Arnaud Chiolero, MD PhD, IUMSP, CHUV
**Study Centre(s):**	CHUV Lausanne

**Statistical Considerations:**	**A) *MySweetHeart Trial:***<br>For the primary analyses, differences in the changes in maternal weight and the EPDS depression symptoms score between inclusion after GDM diagnosis and 1 year postpartum at the end of the study between the 2 groups will be analyzed using linear regression analysis. Analyses will be adjusted for the respective baseline values if there are differences between arms. Variables will be transformed if residuals are not normally distributed. We will include potential confounding variables, if necessary. The potential confounding variables are maternal age, sex of the children, pre-, peri- and early postnatal conditions/complications, and socioeconomic status where applicable.<br>**B) *MySweetHeart Cohort:***<br>The association between exposure (GDM/no GDM) and the (continuous) primary outcomes will be estimated with linear regression analyses, with adjustment for potential confounding factors. We will also conduct similar analyses to assess the association between exposure (GDM/no GDM) and secondary outcomes. Furthermore, the direct and indirect (through mediators) effect of GDM on the primary outcomes will be estimated by causal mediation analyses (notably with an adjustment on birth weight to estimate the indirect effect -not mediated by gestational weight- of GDM on the outcomes).
**GCP Statement:**	This study will be conducted in compliance with the protocol, the current version of the Declaration of Helsinki, the ICH-GCP or ISO EN 14155 (as far as applicable) as well as all national legal and regulatory requirements.

STUDY SUMMARY

Background

Gestational diabetes mellitus (GDM) is a state of glucose intolerance with onset during pregnancy that does not fulfil the criteria for diabetes. It is common with prevalence estimates larger than 10% in several populations. GDM carries pre- and perinatal risk for the mother (e.g., preeclampsia or preterm delivery) and the infant (e.g. macrosomia or neonatal hypoglycaemia) as well as longer-term risks for both the mother (e.g., type 2 diabetes, metabolic syndrome, and cardiovascular disease) and her child (e.g., obesity and type 2 diabetes). Compared to women without GDM, women with GDM are twice as likely to develop perinatal or postpartum depression and approximately one-third of women with recent GDM develop postpartum depression. Lifestyle interventions for the treatment of GDM are often limited to physical activity or nutrition. They often treat the mother or the child separately, and happen either only during or only after pregnancy, and results are inconsistent. The multifactorial origins of GDM and the tight link between mental and metabolic as well as maternal and child health calls for an interdisciplinary approach. Furthermore, maternal GDM may be involved in the fetal programming of long-term cardiovascular health. However, evidence is sparse and the effect of GDM on the child's cardiovascular health is not known.

To address these issues, we will conduct ***MySweetHeart study*** composed of two sub-studies (A) ***MySweetHeart Trial*** (PI: Prof. J. Puder and Dr A. Horsch) and (B) ***MySweetHeart Cohort*** (PI: Prof. N. Sekarski and Dr A. Chiolero). Both sub-studies will be conducted in complete coordination.

A) Objectives of *MySweetHeart Trial:*

To test the effects of a multidimensional interdisciplinary lifestyle and psychosocial intervention to improve/in improving the cardio-metabolic and mental health of women with GDM and their offspring. The primary outcomes are differences between the intervention and the control group in (1) the decrease in maternal weight between 24-32 weeks gestational age and 1 yr postpartum and (2) the decrease in maternal symptoms of depression during the same time period. Maternal secondary outcomes are (1) lifestyle behaviours, aerobic fitness and strength, body composition and cardio-metabolic laboratory biomarkers and (2) other mental health indicators during the peri- and postpartum period. Offspring secondary outcomes are (1) cardio-metabolic laboratory biomarkers at birth, body composition at birth and at 1 yr of age; and (2) mental health indicators at 1 yr of age. Links between these outcomes will also be investigated.

B) Objectives of *MySweetHeart Cohort:*

To assess the effects of GDM on offspring cardiovascular health early in life. The primary outcomes are the differences in surrogate markers of CVD at birth [left ventricular mass index (LVMI) and subclinical atherosclerosis (carotid intima-media thickness; cIMT)] between offspring of women with or without GDM. The secondary outcomes are the differences in cardiovascular structure and function during the foetal period (foetal cardiovascular alterations, LVMI, liver volume), and neonatal adverse cardiovascular risk factors between offspring of women with or without GDM.

Methodology

A) *MySweetHeart Trial:* monocentric superiority open randomized controlled trial (RCT) of 200 women with GDM and their offspring randomly assigned (1:1) to either the intervention (multidimensional interdisciplinary lifestyle and psychosocial intervention) or the control group (treatment-as-usual). Patients will be recruited at 24-32wks of gestation after GDM diagnosis and will be followed-up with their offspring during the first year postpartum. The intervention, offered on top of usual care, consists of individual sessions (face-to-face or telephone contact) with different members of the interdisciplinary team (dietician, physiotherapist, clinical psychologist or coach) and two group sessions. The intervention will consist of: specific dietary and physical activity advice, screening for and treatment of mental health problems, and social support. It will take place during pregnancy and during the first year postpartum. Primary outcomes will be measured at inclusion and 1 year postpartum. Secondary outcomes will be measured at inclusion, 30-34 wks of gestation, and at 6-8 wks and 1 year postpartum. Assessors measuring the primary outcomes and the statistician will be blind to group allocation. For the assessment of outcomes, validated questionnaires and standardised devices, such as calibrated scales, accelerometer, bioimpedance, bone densitometry (*Dual energy X-ray absorptiometry* DXA), and standardised motor tests will be used. Biomarkers and questionnaires will be analysed by staff blind to group allocation.

B) *MySweetHeart Cohort:* cohort study of 100 women with GDM that correspond to the above mentionned control group and their offspring and of 100 women without GDM and their offspring. Patients will be recruited at 24-32 wks of gestation and will be followed-up with their offspring until birth. Primary outcomes will be measured shortly (2-7 days) after birth. Secondary outcomes will be measured during the foetal period (30-34 wks of gestation), at birth, and shortly (2-7 days) after birth. A long-term follow-up of these children is planned but is not part of the current study protocol.

Anticipated results

A) MySweetHeart Trial: We hypothesize that, compared with patients in the control group, patients in the intervention group will have a greater decrease in maternal weight and in maternal symptoms of depression between 24-32 weeks gestational age and 1 yr postpartum. In addition, we anticipate that patients in the intervention group and their offspring will have better cardio-metabolic and mental health outcomes compared to patients and their offspring in the control group.

B) MySweetHeart Cohort: We hypothesize that offspring of women with GDM have a larger LVMI and a larger cIMT at birth (primary outcomes) compared with offspring of women without GDM. Moreover, we hypothesize that offspring of women with GDM will have more foetal cardiovascular alterations and adverse neonatal cardio-metabolic risk factors (secondary outcomes) compared with offspring of women without GDM.

Significance

MySweetHeart Study allows to evaluate if an evidence-based interdisciplinary multimodal lifestyle and psychosocial intervention leads to an improvement in both maternal and offspring cardio-metabolic and mental health outcomes which both represent complications of GDM. If this is the case, this would imply significant changes for clinical practice and guidelines. Furthermore, we will acquire unique insight into the early mechanisms underlying the development of cardiovascular and metabolic diseases, and on the role of mild maternal hyperglycaemic disorders. Having a better understanding of the clinical impact of maternal hyperglycaemic disorders on offspring's health early in life is needed to design a strategy for the early prevention of cardiovascular diseases

Key words

Gestational diabetes mellitus, interdisciplinary, lifestyle intervention, RCT, cohort

ABBREVIATIONS

Provide a list of abbreviations used in the protocol - to be completed

AE	Adverse Event
ASR	Annual Safety Report
BMI	Body mass index
CA	Competent Authority (e.g. Swissmedic)
CEC	Competent Ethics Committee
CIMT	Carotid intima media thickness
CRF	Case Report Form
ClinO	Ordinance on Clinical Trials in Human Research *(in German: KlinV, in French: OClin)*
DM1	Diabetes mellitus type 1
DM2	Diabetes mellitus type 2
eCRF	Electronic Case Report Form
CRP	C-reactive protein
GCP	Good Clinical Practice
GDM	Gestational diabetes mellitus
HAPO	Hyperglycemia and adverse pregnancy outcome
HbA1c	Glycosylated hemoglobin
IB	Investigator’s Brochure
ITT	Intention to treat
LPTh	Loi sur les produits thérapeutiques
LRH	Loi fédérale relative à la recherche sur l’être humain
LVMI	Left ventricular mass index
OClin	Ordonnance sur les essais cliniques dans le cadre de la recherche sur l'être humain *(in German : KlinV, in English : ClinO)*
OGTT	Oral glucose tolerance test
PA	Physical activity
PI	Principal Investigator
SOP	Standard Operating Procedure

Quansah DY, *et al. BMJMED* 2024; 3:e000588. doi: 10.1136/bmjmed-2023-000588

STUDY SCHEDULE

Table 1: Schedule of events

	Timing		NON-GDM N=100	GDM-control N=100	GDM-Intervention N=100
**PREPARTUM**	**24-32**	**GW**	Recruitment	Recruitment	Recruitment
	**24-32**	**GW**	Baseline assessment	Baseline assessment	Baseline assessment
				Usual follow-up	1st session - dietician
					Coach Intervention
	**25-32**	**GW**		Usual follow-up	1st session - physiotherapist
					Coach Intervention
					2nd session - dietician
					Coach Intervention
	**30-34**	**GW**	Fetal cardiac echography	Fetal cardiac echography	Fetal cardiac echography
					2nd session - physiotherapist
					Coach Intervention
	**33-38**	**GW**		Follow-up	3rd session - dietician
					Coach Intervention
					Pre-natal workshop
					Follow-up and assessment
**CHILDBIRTH**	**Birth**		Cord blood (biomarkers) & maternal blood	Cord blood (biomarkers) & maternal blood	Cord blood (biomarkers) & maternal blood
**POSTPARTUM**	**2-7 days**	**PP**	Cardiac echography and carotid dupler	Cardiac echography and carotid dupler	Cardiac echography and carotid dupler
	**6-8 wks**	**PP**		Follow-up and assessment	Follow-up and assessment
	**3 mo**	**PP**			Post-partum workshop
	**5 mo**	**PP**			Interdisciplinary session
	**8 mo**	**PP**			Interdisciplinary session
	**10 mo**	**PP**			Interdisciplinary session
	**12 mo**	**PP**		Follow-up and assessment	Follow-up and assessment

Table 2: Schedule of assessments. All these variables are measured among women (and their offspring) with GDM. Variables also measured among women (and their offspring) without GDM of *MySweetHeart Cohort* are indicated by a star (*).

				TIMING						
	**DOMAIN**	**VARIABLES**	**INSTRUMENTS**	**24-32 GW**	**30-34 GW**	**33-38 GW**	**Birth**	**2-7 d PP**	**6-8 wks PP**	**1 yr PP**
**MOTHER**	**Physical exam**	Anthropomorphic measures*: Pregravid weight, gestational weight gain, weight retention, BMI	Height, weight	X		X			X	X
		Total fat mass	Bioimpedance, skinfolds (calipers)	X					X	X
		Total and regional fat mass	Dual-Energy X-Ray absorptiometry (Lunar®)							X
	**Sociodemographic background**	Sociodemographic variables*, health literacy*, exposure to life events*	Sociodemographic questionnaire, health literacy question, Life Events Questionnaire	X						X
	**Lifestyle behaviours**	Carbohydrate and fat intake	Food Frequency Questionnaire	X						X
		Eating behaviour	French Intuitive Eating Scale (routine 1st visit)	X						X
		Breastfeeding	Self-report (duration and exclusiveness)						X	X
		Feeding behaviours:								
		Food to soothe	Food to Soothe Questionnaire							X
		Hunger/satiety clues	Infant Feeding Style Questionnaire: Satiety subscale							X
		Physical activity	Accelerometer (GeneActiv®): Total counts/mn & time spent in moderate-vigorous and sedentary activity.	X						X
		Sleep quality and quantity	Pittsburgh Sleep Quality Index	X						
		Aerobic fitness	Chester step test with VO2max estimation	X						X
		Strength	Prehension: grip strength (Jamar dynamometer)	X						X
	**Mental Health**	Depression*	Edinburgh Postnatal Depression Scale (routine)	X					X	X
			Whooley questions (routine)	X					X	X
		Anxiety*	Anxiety subscale of Hospital Anxiety and Depression Scale	X						X
		Well-being*	WHO Well-Being Index (routine)	X		X			X	X
		Parenting stress	Parenting Stress Scale-short form							X
		Social support*	Medical Outcomes Study Social Support Survey-short form	X						X
		Life events*	Life Events Questionnaire	X						X
	**Cardiometabolic laboratory biomarkers**	mi-RNA (in plasma)*	plasma; various mi-RNA	X			X			X
		Overall metabolic control	HbA1c (routine)	X		X			X	X
		Glucose tolerance	Oral glucose tolerance test (routine)						X	X
		Cardiovascular and metabolic risk markers*	see laboratory xls	X					X	X
**PARTNER**	**Physical exam**	Anthropomorphic measures	Height, weight (measured) and BMI	X						X
	**Sociodemographic background**	Sociodemographic variables, health literacy, exposure to life events	Sociodemographic questionnaire, health literacy question, Life Events Questionnaire	X						
	**Lifestyle behaviours**	Eating behaviour	French Intuitive Eating Scale 2	X						X
	**Mental Health**	Well-being	WHO Well-Being Index	X						X
		Depression	Edinburgh Postnatal Depression Scale	X						X
		Anxiety	Anxiety subscale of Hospital Anxiety and Depression Scale	X						X
		Parenting stress	Parenting Stress Scale-short form							X
		Social support	Medical Outcomes Study Social Support Survey-short form	X						X
**CHILD**	**Physical exam**	Anthropomorphic measures	Height, weight (calibrated baby scale), BMI				X		X	X
		Total fat mass	Bioimpedance							X
			Skinfolds (calipers)						X	X
	**Mental health**	Self-regulation	Difficult Child' subscale of Parenting Stress Index-short form							X
		Sleep quality and quantity	Brief Infant Sleep Questionnaire							X
	**Cardiometabolic laboratory biomarkers**	mi-RNA*	Cord blood; various mi-RNA				X			
		Cardiovascular and metabolic risk markers*	Cord blood; glucose, HbA1C, insulin, total cholesterol, HDL-cholesterol, triglycerides, uric acid, creatinine, hs-CRP				X			
	**Cardiac health**	Blood pressure*	Systolic and diastolic blood pressure	X	X			X		
		Cardiac structure and function*	Fetal ultrasound: cardiac structure and function and liver size		X					
		Cardiac and carotid structure and function*	Cardiac (LVMI) and carotid ultrasound (cIMT)					X		

1. STUDY ADMINISTRATIVE STRUCTURE

1.1. Sponsor, Sponsor-Investigator
Prof Jardena Puder, MD, Service d'endocrinologie, CHUV

1.2. Principal Investigator(s)
A) *MySweetHeart Trial* :
Prof. Jardena Puder, MD, Service d'endocrinologie, CHUV and
Dr Antje Horsch, PhD, Département Femme, Mère & Enfant, CHUV
B) *MySweetHeart Cohort* :
Prof. Nicole Sekarski, MD, Unité de Cardiologie pédiatrique, CHUV and
PD & MER Arnaud Chiolero, MD PhD, IUMSP, CHUV

1.3. Statistician ("Biostatistician")
PD & MER Arnaud Chiolero, MD PhD, IUMSP, CHUV

1.4. Laboratory
CHUV Laboratory (head: Dr Daniel Bardy)

1.5. Monitoring institution
CHUV

1.6. Data Safety Monitoring Committee
There is no safety monitoring committee because the intervention is not associated with any substantial risk for the participants (see below point 5.4. Safety outcomes).

1.7. Any other relevant Committee, Person, Organisation, Institution
Not applicable.

2. ETHICAL AND REGULATORY ASPECTS

2.1. Study registration
MySweetHeart Trial will be registered in clinicaltrials.gov and kofam.ch after the positive decision of the CER-VD has been received.

2.2. Categorisation of study
A) *MySweetHeart Trial:* category A, as it entails only minimal risks and burdens. The intervention consists of a multidimensional interdisciplinary lifestyle and psychosocial intervention and no medicinal product will be given.
B) *MySweetHeart Cohort:* category A as it entails only minimal risks and burdens.

2.3. Competent Ethics Committee (CEC)
CER-VD

2.4. Competent Authorities (CA)
Not applicable

2.5. Ethical Conduct of the Study
The study will be carried out in accordance with the protocol and with principles enunciated in the current version of the Declaration of Helsinki, the guidelines of Good Clinical Practice (GCP) issued by ICH, in case of medical device: the European Directive on medical devices 93/42/EEC and the ISO Norm 14155 and ISO 14971, the Swiss Law and Swiss regulatory authority's requirements.

2.6. Declaration of interest
The investigators declare no conflict of interest.

2.7. Patient Information and Informed Consent
The investigators will explain to each participant the nature of the study, its purpose, the procedures involved, the expected duration, the potential risks and benefits and any discomfort it may entail.

Each participant will be informed that the participation in the study is voluntary, that she may withdraw from the

study at any time, and that withdrawal of consent will not affect her subsequent medical assistance and treatment or that of her child. Each participant will be provided with an information sheet and a consent form describing the study and providing sufficient information to make an informed decision about their participation in the study. The formal consent of a participant, using the approved consent form, will be obtained before the participant is submitted to any study procedure. The participant should read and consider the statement before signing and dating the informed consent form, and should be given a copy of the signed document. The consent form must also be signed and dated by the investigator (or his designee) and it will be retained as part of the study records. After a potential participant has been given the information sheet, she will have a minimum of 24 hours to consider her participation in the study.

A) *MySweetHeart Trial*: participants will receive for their time, effort, and travel costs CHF 250 at 6-8 weeks postpartum and CHF 200 at 1 year postpartum after the respective assessments. Partners of GDM patients will also be recruited after agreement of the patients and will receive CHF 50 at 1 year postpartum as compensation for their time, effort, and travel costs.

B) *MySweetHeart Cohort*: participants will receive CHF 100 as compensation for their time, effort, and travel costs.

Participant privacy and confidentiality
The investigators affirm and uphold the principle of the participant's right to privacy and that they shall comply with applicable privacy laws. In particular, anonymity of the participants shall be guaranteed when presenting the data at scientific meetings or publishing them in scientific journals.

Confidentiality will be ensured by utilising subject identification code numbers to correspond to treatment data in the computer files. Furthermore, study data will be stored in a password-protected database (Secutrial) and any paper records relating to the study will be kept in a locked filing cabinet.

For data verification purposes, authorised body such as the ethics committee may require direct access to parts of the medical records relevant to the study, including participants' medical history.

2.8. Early termination of the study
A) *MySweetHeart Trial:* The investigators may terminate the trial prematurely according to certain circumstances, for example: insufficient participant recruitment, when alterations in accepted clinical practice that make the continuation of a clinical trial unwise, or early evidence of benefit or harm of the experimental intervention.
B) *MySweetHeart Cohort:* Not applicable

2.9. Protocol amendments
Substantial amendments are only implemented after approval of the CEC respectively. Only the PI and team of co-investigators are allowed to provide suggestions for a protocol amendment. Under emergency circumstances, deviations from the protocol to protect the rights, safety and well-being of human subjects may proceed without prior approval of the PI and the CEC. Such deviations shall be documented and reported to the PI and the CEC as soon as possible. All non-substantial amendments are communicated to the CEC within the Annual Safety Report (ASR).

3. BACKGROUND AND RATIONALE

3.1. Background

A) *MySweetHeart Trial:*
Our objective is to test the effect of a multidimensional interdisciplinary lifestyle and psychosocial intervention to improve the cardio-metabolic and mental health of women with GDM and their offspring.

Definition of gestational diabetes mellitus (GDM)
GDM is characterized by glucose intolerance diagnosed during pregnancy that does not fullfill the criteria for diabetes and usually resolves after delivery. The "Hyperglycemia and adverse pregnancy outcome" (HAPO)[1] study found strong associations of maternal glucose levels with increased birth weight and cord-blood C-peptide levels, as well as with primary cesarean delivery, pre-eclampsia and pre-term delivery. The HAPO study led to more stringent diagnostic GDM criteria and based on the study results, a 75-g oral glucose-tolerance test (OGTT) at 24-28 weeks of gestation is recommended [2, 3]. The prevalence of GDM in the HAPO study (17.8%) and elsewhere (10-15% in our tertiary setting at the CHUV hospital and 10.9% in the Geneva and Basel Hospitals [4] is linked to the high prevalence of obesity and physical inactivity, as well as the new screening

recommendations and criteria to diagnose GDM.

Morbidity of GDM and related conditions

GDM carries pre- and perinatal risk for the *mother* (pre-eclampsia, primary cesarean delivery, preterm delivery, hydramnios) and the child (macrosomia, neonatal hypoglycemia, birth injury, respiratory problems, hyperbilirubinemia, hypocalcemia, intensive care). It also carries long-term maternal risks such as a 30-70% GDM recurrence, a 7-fold higher 5-10 yr risk of type 2 diabetes (DM2), or an increased risk of metabolic syndrome and cardiovascular disease [5-8]. Even mild glucose intolerance in pregnancy is related to a 70% higher relative risk of cardiovascular disease over a 12 year median follow-up [7]. Compared to women without GDM, women with GDM are twice as likely to develop perinatal or postpartum depression and approximately one-third of women with recent GDM develop postpartum depression [9]. Postpartum depression leads to a decrease in physical activity and comfort eating, thus putting the women at higher risk of weight gain and future diabetes. It also leads to a decline in effective parenting skills, which may have negative consequences for the development of the child. Indeed, many parents of obese children lack effective parenting skills that provide both a consistent structured frame and emotional support, and thus effective prevention strategies are needed.

In women with GDM, psychosocial vulnerability including low levels of social and family networks is associated with increases in adverse outcomes, especially infants' increased birth weight [10]. On the other hand, social support in women with GDM can promote diabetes self-care during pregnancy [11] and is a key factor for PA and a healthy diet and breastfeeding in the postpartum period [12-14]. Social support is also a key factor in improving mental health during pregnancy [15].

Regarding the child, the importance of the intrauterine and early postnatal environments for metabolic programming and modifications of the epigenome is increasingly recognized [16]. Within a Developmental Origin of Health and Disease (DOHaD) framework [17, 18], fetal programming is the process involved in the associations between the exposure to detrimental factors during fetal life and health outcomes, particularly metabolic diseases, later in life [19]. Thus, there is an independent relationship between GDM exposure with macrosomia at birth and with (central) obesity and insulin secretion in children, while the relationship with obesity in infants is more controversial and seems to relate to maternal BMI or birth weight [20-24]. Intrauterine exposure to GDM also doubles the risk for subsequent DM2 in offspring compared to offspring of mothers with a high genetic predisposition for DM2, but with normal glucose tolerance during the index pregnancy [25].

Among epigenetic changes that are modified by the environment, micoRNAs (miRNAs) are getting increasing interest. MiRNAs are single-stranded non-coding RNAs of approximately 21–23 nucleotides in length whose main function is to inhibit gene expression by interfering with mRNA processes. MiRNAs suppress gene expression by affecting mRNA stability, targeting the mRNA for degradation, or both [26]. Nearly 1,000 miRNAs have been identified in human cells with the potential to regulate the expression of about two-third of human mRNAs and influence almost all genetic pathways including metabolism [27]. Animal maternal obesity and changes in diet lead to altered expression of microRNA in offspring, influencing gene expression [26, 28, 29]. Thus, miRNA's may have a critical role in the programming of metabolic alterations induced by in utero exposure to high fat diet. For instance, Fernandez-Twinn et al. showed a decrease in IRS-1 protein in adipose tissue of offspring of obese mice, correlated with miR-126 (which targets IRS-1) increase [30]. Also, the differential effects of maternal obesity and weight loss in the periconceptional period on the hepatic insulin-signaling pathways in the offspring have been linked to alterations in 29b, miR-103, and miR-107 expression [31].

Maternal pre-pregnancy overweight and excessive gestational weight gain also predict high birth weight and adiposity during infancy [16, 32]. This is highly relevant, as up to 60-70% of women with GDM are overweight or obese before pregnancy [33]. Maternal obesity during pregnancy is associated with increased hospital admission for cardiovascular events, increases the risk of adverse outcomes and all cause mortality in offspring independent of confounders [34]. Finally, lifestyle behavior such as a high fat diet or physical activity during pregnancy can influence offspring adiposity independent of maternal obesity [16, 35]. Recent evidence shows an increased risk of diabetes in *partners* of mothers with GDM, which is partly mediated by shared deprivation level and cultural background as well as lifestyle behavior [36].

The link between maternal, child, and paternal metabolic health creates a deleterious vicious cycle in view of the huge and increasing worldwide prevalence of (childhood) obesity and subsequent metabolic problems despite national and international intervention efforts [37]. ***Because of the deleterious impact of GDM and lifestyle during pregnancy on the health of the mother and her offspring, it is crucial to intervene during the critical window of the pre-, peri-and postnatal period.***

Modifiable risk factors of GDM

Figure 1 presents the modifiable risk factors of GDM that our planned intervention will address. Physical activity is a modifiable determinant of GDM. During pregnancy, physical activity is protective and reduces the risk of

GDM [38]. It decreases insulin resistance and limits gestational weight gain by increasing energy expenditure and altering food intake [39]. Therefore, physical *in*activity is a potent risk factor. Nutrition is another major and modifiable determinant of GDM. High fat consumption increases GDM risk, especially saturated fat, trans fat and cholesterol [40]. Animal protein intake is positively and vegetable protein inversely associated with GDM risk [41].

Another key determinant is mental health. Higher stress exposure during pregnancy has been reported in women with GDM compared to those without [42, 43]. Our recent prospective study in 221 women showed that higher stress exposure and perceived stress were associated with increased fasting glucose levels before women knew their diagnosis [44]. Psychological stress and negative life events are associated with higher salivary cortisol levels during pregnancy, which might influence glucose levels [45]. On the other hand, good social support has been shown to be protective regarding mental health and depression in particular. Social support is also associated with increased PA in women who have a high risk of GDM [46]. In addition, women with GDM report to have less social support [47].

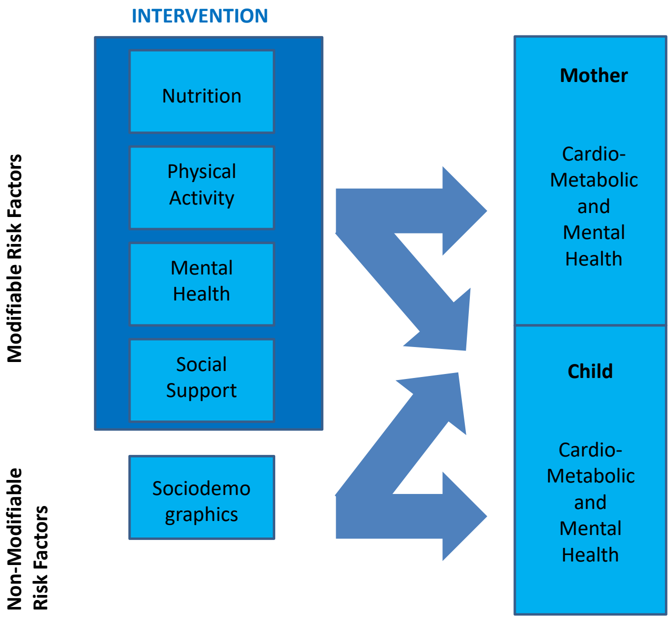

Figure 1: Modifiable risk factors of GDM that will be targeted by the multidimensional interdisciplinary lifestyle intervention

Prior studies having evaluated lifestyle interventions in GDM

Generally, interventions in GDM aim for dietary and/or physical activity changes, focusing either on pregnancy or the postpartum (except for one research group [48, 49]) and only on the mother [48, 49]. So far, their effects are limited and concern only few health aspects.

Dietary advice is recommended for all women with GDM to improve glycemic control and to provide adequate nutrition [3]. Of the few existing trials, almost all focussed on either low carbohydrate or low glycemic index foods, but either type of intervention did not consistently beneficially impact on glycemic values, despite some favourable impact on insulin requirement and/or maternal weight gain [50, 51]. A 2013 Cochrane review found insufficient evidence for strong conclusions about what dietary advice to give to women with GDM [52]. As fat, especially saturated fat, is a risk factor for both GDM and DM2, decreasing animal fat intake represents an interesting novel approach [40, 53-55]. Indeed, a higher-complex carbohydrate/low-fat diet including complex carbohydrates improved glycemic values, insulin resistance in women with GDM as well as infant adiposity [56].

Although recommended for GDM treatment, guidelines do not specify the type of physical activity or its timing in regards to meal intake [3, 57]. Resistance and endurance exercise can be accomplished during pregnancy in the absence of contraindications, but motivation and compliance appear to be major limiting factors [58]. A recent review concluded that physical activity may improve glycemic control and/or limit insulin use in women with GDM[59]. Regular physical activity can also limit pregnancy weight gain, stabilize maternal mood, and limit fetal fat mass and physiological stress responses in the offspring [35, 58, 60].

To date, there are no evidence-based psychological interventions for women with GDM and no international guidelines addressing their psychosocial management. Recent research has looked at psychological interventions such as mindfulness-based eating awareness that aims at increasing awareness of inner cues, such as hunger and satiety, at identification of emotional eating and eating triggers, and at improving self-

acceptance. One study applied mindfulness eating and yoga exercise and reported reduced fasting plasma glucose, 2-h postprandial blood glucose (of around 0.5 mmol/l), and a reduction in HbA1c of 0.5% [61].

Postpartum management and follow-up
Due to the increased risk of persistence or development of prediabetes and diabetes, management of women with GDM in the postpartum period is essential and should focus both on regular screening and on prevention strategies. The American Diabetes Association, the American College of obstetricians and Gynecologists and the National Diabetes Education Program recommend testing within 6-12 weeks postpartum with a one step 2hr 75-g OGTT[57, 62, 63]. Due to the low attendance rate at postpartum OGTT screening (34 to maximally 73% in published series) there is a need to simplify the follow-up of women with previous GDM[64]. The highest risk period for the development of DM2 is within the first 5 years after a GDM pregnancy[65]. Despite this critical timeframe, there is a scarcity of RCT's in this young population[65].

Weight loss is an important predictor to prevent diabtetes in high-risk population. Thus, in the Diabetes Prevention Program DPP weight loss after GDM reduced future diabetes incidence by 16% for every kilogram lost [66]. Existing large randomised studies in women after GDM lead to a decrease in weight, while the impact on diabetes incidence was controversial [66-69].

The "Diet, Exercise and Breastfeeding Intervention" (DEBI) study [48] is the only study that started during pregnancy and continued postpartum, and *was mostly delivered by phone. The proportion of women who reached their 1 year postpartum weight goal tended to be higher in the intervention arm (37.5 vs 21.4%, p=0.07).* However, to our knowledge, no large post-GDM RCTs exist in a multi-ethnic European population.

Prevention strategies in the child
Offspring of women with GDM are at higher risk for childhood obesity. Several modifiable risk factors have been identified, such as infant feeding mode (bottle vs breastfeeding), infant sleep duration, parental reaction to regulate infant distress and (particularly nocturnal) crying, timing of the introduction of solid food, sweetend beverage consumption, and the age of bottle weaning[70]. For example, breastfeeding attenuates the increased risk of childhood adiposity that is associated with exposure to diabetes during pregnancy[71]. Feeding is often used as a first response to infant distress[72] and is more prevalent in mothers who eat themselves when distressed[73]. Parents with overweight, obesity or diabetes may adapt controlling child-feeding practices and these parental control attempts may interact with genetic predispositions to promote the development of problematic eating styles and childhood overweight. A parenting skills intervention should focus on these factors, provide anticipatory guidance and teach parents how to identify and respond appropriately to infant cues and distress to positively influence self-regulatory capacities, well-being and the developing control of the infant's food intake in order to avoid eating in the absence of hunger[74, 75].

Development of a multidimensional interdisciplinary lifestyle intervention
Given that single risk factor interventions have shown limited efficacy, multidimensional approaches targeting both the mother and the child and including the partner could be more efficient considering the complex multifactorial origin of GDM and its transgenerational importance [76] **(Figure 1)**. Indeed, a multidimensional interdisciplinary and psychosocial approach integrating the above mentioned modifiable risk factors in a concerted and individually adapted fashion could help to improve the treatment of GDM and to reduce long-term complications [77]. Such an approach would need to be started in pregnancy and continue postpartum.

In summary, the multifactorial origin of GDM, and the close link between the different risk factors as well as between maternal, child, and paternal health and the critical window of the perinatal period ***call for a multidimensional interdisciplinary lifestyle and psychosocial interventioninterdisciplinary that spans through pregnancy and the postpartum period and focuses on the health of the mother and her child***.

B) *MySweetHeart Cohort:*
Cardio-metabolic effects of GDM within a DOHaD framework
Maternal hyperglycemic disorders are associated with offspring's fetal cardiac and vascular structural and functional alterations. For instance, it is well known that offspring of T1/T2DM mothers are at increased risk of congenital cardiac malformations and cardiac hypertrophy [78, 79]. In a retrospective study of 92 offspring of 87 diabetic mothers conducted in Lausanne, we observed that 5 neonates had congenital heart disease and 12 had ventricular hypertrophy [78]. There is also growing evidence that maternal hyperglycemic disorders may be involved in the "fetal programming" of obesity and of metabolic disorders in their offspring [80]. Within a Developmental Origin of Health and Disease (DOHaD) framework [81, 82], fetal programming is the process involved in the associations between the exposure to detrimental factors during fetal life and health outcomes later in life [83]. Fetal programming is suspected to be involved in the development of cardio-metabolic disorders, such as elevated blood pressure, coronary heart diseases or DM, notably through epigenetic mechanisms [81, 82]. Despite recent and large growth in this research area, studies are needed to better objectively characterize both early life exposure and cardio-metabolic outcomes [84].

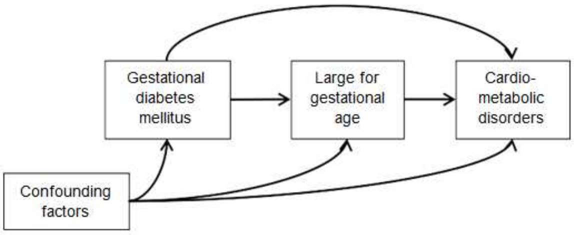

Figure 2: Hypothetical and highly-simplified causal relationships between gestational diabetes mellitus (GDM) and cardio-metabolic disorders. Two pathways can be hypothesized: 1) a direct effect of GDM on offspring's cardio-metabolic disorders; 2) an indirect effect through the mediator large for gestational age (LGA). It means that if LGA was prevented, part of the effect of GDM on cardio-metabolic disorders would be prevented. Several confounding factors (such as maternal obesity, weight gain during pregnancy, smoking, socio-economic status, or family history of cardio-metabolic diseases) have to be accounted for.

Several epidemiologic studies suggest that intrauterine environment of women with DM contribute to long term risk of metabolic diseases and intergenerational transmission of cardio-metabolic risk [80]. For example, adult offspring of women with DM during gestation have increased risk of overweight/obesity [85-88], metabolic syndrome [85, 87], T2DM [89], or pre-diabetes [89]. Exposure to maternal impaired glucose tolerance or DM during fetal life is associated with large fetal fat mass [90] and overweight/obesity in childhood [91]. A large sibling study shows that the association between maternal DM and offspring BMI was most likely due to in utero exposure to hyperglycemia and not to confounding by shared familial characteristics [92]. It was also shown that offspring who had been exposed to maternal DM during fetal life exhibit higher prevalence of impaired glucose tolerance and markers of insulin resistance in childhood and adolescence [93, 94]. One study shows that LGA offspring of GDM mothers are at greater risk of developing metabolic syndrome disorders compared to non-LGA offspring of GDM mothers [95]. LGA may be therefore a mediator on the causal pathway between maternal hyperglycemic disorders, including GDM, and cardio-metabolic disorders, and hence could be a clinical marker of fetal programming **(Figure 2)**. The association between maternal hyperglycemic disorders and LGA seems to be due to the increase in fetal insulin production in response to the increased transplacental transfer of maternal glucose [96, 97]. In the HAPO study, maternal hyperglycemia and fetal hyperinsulinism were associated with high birth weight. Intrauterine exposure to high maternal blood glucose has also been associated with greater lean mass and adiposity among prepubertal offspring [98].

During fetal life, maternal diabetes is associated with fetal hyperinsulinism responsible of structural and functional change affecting mostly the liver and the cardiovascular system. The liver is the major metabolic organ, considered as the metabolic brain and responsible for the distribution of the placental resources [99]. Recent studies have shown that macrosomic offspring had impaired liver blood perfusion that contributes to the dysregulation of the fetal growth [100-102] and that differential perfusion of the fetal liver modified hepatic metabolic function and size [99]. Studies indicate that the liver is enlarged in fetuses of T1/T2 DM mothers [103, 104]. Furthermore, and similarly, the fetal heart response to hyperinsulinisim is the development of an asymmetrical hypertrophy predominant at the septal wall. Cardiac hypertrophy results rarely in major cardiac outflow obstruction. Usually, by 6 months of age, it has totally resolved without further long-term functional repercussion [105]. ***The evaluation of liver volume, interventricular septum thickness, and left ventricular mass (LVM) is therefore of outmost importance and may represent surrogate markers of the fetal metabolic response to maternal GDM.***

Lack of data on the effects of GDM on offspring's cardiovascular health
Most studies on the effect of maternal hyperglycemic disorders were conducted among mothers with T1/T2DM, not among mothers with GDM, and with an emphasis on the effects on offspring's metabolic outcomes rather than on offspring's cardiovascular health. It is also unknown whether treatment of GDM is effective to reduce long-term risk of offspring's metabolic and CVD risk [80, 106, 107] and the new definition of GDM calls also for the conduction of further studies on the impact of GDM on offspring's cardiovascular health. To study the early development of atherosclerosis and pathogenesis of CVD, it has become central to assess surrogate markers of CVD such as increased cardiac mass (left ventricular mass index; LVMI) [108] and of subclinical atherosclerosis (assessed trough carotid intima-media thickness; cIMT) [108, 109]. These markers have been used extensively in studies in children and young adults with CVD risk factors [108-110]. ***However, to our knowledge, the effects of GDM on fetal and early neonatal offspring's cardiovascular health, and in particular on such surrogate markers, have never been studied.***

3.2. Investigational Product (treatment, device) and Indication
Not applicable

3.3. Preclinical Evidence
Not applicable

3.4. Clinical Evidence to Date

A) *MySweetHeart Trial*:

We have conducted several pilot studies that form the base for the rationale of the multidimensional intervention.
(1) Our study on stress and GDM [44] confirmed the rationale to integrate a psychosocial part in the intervention, as we showed for the first time that higher stress exposure and higher perceived stress were associated with higher fasting glucose levels before women knew their diagnosis.
(2) A clinical GDM cohort study of 322 patients that we started in 2013, helped us to (1) assess the prevalence of overweight (27%) and obesity (22%; which is 3-fold higher compared to women of the large Co-Laus cohort in the same city, but lower than in other studies, (2) to assess the mean gestational weight gain, the postpartum weight retention and symptoms of postpartum depression.
(3) Two focus groups of our GDM patients suggested feasibility (home-based exercises, need for support) and identified barriers (having young children, lack of risk perception, anxiety) of our planned intervention.
(4) We also performed a small postpartum physical activity study over 3 months in which women in the intervention arm significantly increased their reported physical activity (p=0.03) and lost 2.3 ± 1.4 kg more weight (*p* = 0.1) than women in the control arm.

B) *MySweetHeart Cohort:*

We have conducted a study to insure that the measurement of carotid intima-media thickness (CIMT), our primary outcome, was feasible in young non-sedated infants (Mivelaz et al, under review). This study conducted among 81 infants less than 1 years of age confirmed that CIMT was measurable with a high inter-observer reliability (coefficient of variation: 5.9%).

3.5. Explanation for choice of comparator (or placebo)

A) *MySweetHeart Trial*:

The comparison group will receive treatment as usual (not placebo), which is based on the current guidelines of the American Diabetes Association [2] and the Endocrine Society [3] (see section 8.2.2. for more details).

B) *MySweetHeart Cohort:*

Not applicable.

3.6. Risks / Benefits

A) *MySweetHeart Trial*:

For the participants allocated to the intervention group, there will be a potential direct benefit from a multidisciplinary lifestyle and psychosocial intervention that may be superior to the current treatment as usual by leading to reduced weight and less symptoms of depression. A potential risk is the occurrence of early contractions due to intense physical activity. In order to monitor and mitigate this potential risk, patients will be closely supervised by a physician and physiotherapist. In case of early contractions, participants will be requested to reduce or stop their physical activity.

For the participants allocated to the control group, there are no anticipated risks or benefits.

B) *MySweetHeart Cohort:*

There are no anticipated risks or benefits. If cardiac anomalies are identified during cardiac echography (but not revealed by the routine prenatal ultrasound), the parents will be informed and counselled by the paediatric cardiologist in the same manner as would be done if the finding had been detected on usual prenatal screening.

3.7. Justification of choice of study population

This study aims at investigating pregnant women (aged >18 years) with GDM or without GDM and their offspring. The choice of pregnant women is linked to the health problem under investigation, GDM being a condition only occurring during pregnancy.

4. STUDY OBJECTIVES

A) *MySweetHeart Trial:*

A4.1) Overall Objective

To test the effect of a multidimensional interdisciplinary lifestyle and psychosocial intervention to improve the cardio-metabolic and mental health of women with GDM and their offspring.

A4.2) Primary Objective

To test the effect of a multidimensional interdisciplinary lifestyle and psychosocial intervention in women with GDM to improve 1) their metabolic (decrease in maternal weight between 24-32 weeks gestational age and 1 yr postpartum) and 2) their mental (decrease in maternal symptoms of depression during the same time period) health.

A4.3) Secondary Objectives

To test the effect of a multidimensional interdisciplinary lifestyle and psychosocial intervention to improve other cardio-metabolic and mental health markers in women with GDM and their offspring.

A4.4) Safety Objectives
Not applicable

B) *MySweetHeart Cohort:*
B4.1) Overall Objective
To assess the effect of GDM on offspring cardiovascular health early in life
B4.2) Primary Objective
To assess the effect of GDM on the surrogate markers of cardiovascular disease (CVD) at birth (left ventricular mass index and subclinical atherosclerosis)
B4.3) Secondary Objectives
To assess the effect of GDM on the cardiovascular structure and function during the fetal period and neonatal adverse cardiovascular risk factors
B4.4) Safety Objectives
Not applicable

5. STUDY OUTCOMES

A) *MySweetHeart Trial:*
A5.1. Primary Outcomes
The primary outcomes are differences between the intervention and the control group in 1) the decrease in maternal weight between inclusion after GDM diagnosis and maternal weight at 1 yr postpartum and 2) the decrease in maternal symptoms of the Edinburgh Postnatal Depression score (EPDS) during the same time period.
A5.2. Secondary Outcomes
Differences between the intervention and the control group in other maternal secondary outcomes, such as (1) lifestyle behaviours, aerobic fitness and strength, body composition and cardio-metabolic laboratory biomarkers and (2) other mental health indicators during the peri- and postpartum period and offspring secondary outcomes, such as (1) cardio-metabolic laboratory biomarkers at birth, body composition at birth and at 1 yr of age; and (2) mental health indicators at 1 yr of age.
A5.3. Other Outcomes of Interest
Not applicable.

A5.4. Safety Outcomes
A potential risk is the occurrence of early contractions due to intense physical activity. In order to monitor and mitigate this potential risk, patients will be closely supervised by a physician and physiotherapist. In case of early contractions, participants will be requested to reduce or stop their physical activity. Any information related to safety issues will be recorded in the CRF.

B) *MySweetHeart Cohort:*
B5.1. Primary Outcomes
Differences in surrogate markers of CVD at birth [left ventricular mass index (LVMI) and subclinical atherosclerosis (carotid intima-media thickness; cIMT)] between offspring of women with and offspring of women without GDM.
B5.2. Secondary Outcomes
Differences in cardiovascular structure and function during the fetal period (fetal cardiovascular alterations, LVMI, liver volume) and in neonatal adverse cardiovascular risk factors between offspring of women with and offspring of women without GDM.
B5.3. Other Outcomes of Interest
Not applicable.
B5.4. Safety Outcomes
Not applicable.

6. STUDY DESIGN

5.1. General study design and justification of design

A) *MySweetHeart Trial:*
Design: monocentric superiority open RCT with minimal risk (category A) aiming to test the effect of a multidimensional interdisciplinary lifestyle and psychosocial intervention for pregnant women with GDM compared with treatment-as-usual.
Population: 200 pregnant women with GDM, their partners, and their offspring.
Study duration: 4 years
Duration of patient's participation: 15 months

Intervention: interdisciplinary multimodal lifestyle and psychosocial intervention (see 8.2.1. for more details).
Comparator: treatment-as-usual (see 8.2.2. for more details).
Recruitment: Patients will be recruited at 24-32 wks of gestation and their offspring will be followed-up during the first year postpartum. The intervention will take place during pregnancy and during the first year postpartum.
Timing of outcome measures: Primary outcomes will be measured at 24-32 wks of gestation and at 1 year postpartum. Secondary and other outcomes will be measured at 30-34 wks of gestation, at birth, 6-8 wks and 1 year postpartum.
Blinding: assessors measuring the primary outcomes and the statistician are blinded to group allocation.
Potential limitations: risk of bias due to (1) lack of blinding of patients and clinicians; (2) contamination due to improvement of usual care by health care providers; (3) selection at the point of recruitment; and (4) loss to follow-up. To limit the impact of these potential biases, we will (1) ensure that the assessors of the primary outcomes and the statistician are blinded to group allocation; (2) monitor any changes in the usual care and account for these changes in the analysis; (3) limit our exclusion criteria to a minimum and widen access by also recruiting from clinics and private practices other than the CHUV; and provide all study documents in English and in French; and (4) pay patients for their participation time and effort and their travel expenses, stay in regular contact with patients, and conduct intention-to-treat analyses.

B) *MySweetHeart Cohort:*
Design: cohort study comparing the cardiovascular health in offspring of pregnant women with GDM and non-GDM.
Population: 100 pregnant women with GDM and 100 pregnant women with GDM and their offspring.
Study duration: 3 years
Duration of participant's participation: 3-4 months
Exposure: GDM vs no GDM.
Recruitment: Patients will be recruited at 24-32 wks of gestation.
Timing of outcome measures: outcomes will be measured at 30-34 wks of gestation and at birth.
Blinding: the assessors measuring the outcomes and the statistician will be blinded to the exposure status.
Potential limitations: risk of bias due to (1) no blinding of patients and clinicians and (2) selection at the point of recruitment. To limit the impact of these potential biases, we will (1) ensure that the assessors of the outcomes and the statisticians are blinded to exposure status and (2) limit our exclusion criteria to a minimum and widen access by also recruiting from clinics other than the CHUV; and provide all study documents in English and in French.

6.2. Methods of minimising bias

6.2.1. Randomisation

A) *MySweetHeart Trial:* The allocation ratio of randomisation is 1:1, using the block randomisation method (blocks of 4) after stratification for the place of the usual care (CHUV vs each private diabetologist). For allocation of the participants, a computer-generated list of random blocks is used. The allocation sequence will be concealed from the research staff assessing the primary outcomes in sequentially numbered, opaque, sealed envelopes. Envelopes will be opened only after the enrolled participants completed all baseline assessments.
B) *MySweetHeart Cohort:* not applicable

6.2.2. Blinding procedures

A) *MySweetHeart Trial:* Assessors of primary outcomes and the statistician will be blind to group allocation.
B) *MySweetHeart Cohort:* Assessors of primary outcomes and data analysts will be blind to exposure.

6.2.3. Other methods of minimising bias

We will use validated questionnaires and devices with standardised norms, such as calibrated scales, accelerometer, bioimpedance, DXA, cardiac and carotid ultrasound, and standardised motor tests. Biomarkers will be analysed by staff blind to group allocation.

6.3. Unblinding Procedures (Code break)

Not applicable.

7. STUDY POPULATION

7.1. Eligibility criteria

Inclusion criteria: Women aged 18 yrs or older, with or without GDM at 24-32 weeks of gestation, and understanding French or English.

Exclusion criteria: Women on strict bed-rest, with pre-existing diabetes or known severe mental disorder

7.2. Recruitment

All pregnant women attending their antenatal appointment at the policlinique at the CHUV for their routine screening for GDM at around 22-31 weeks will be pre-informed about the study (flyer). We will recruit 200 women with GDM and 100 women without GDM.

Women with GDM: Pregnant women who received the diagnosis of GDM are referred to the GDM consultation at the CHUV (expected N = around 250 GDM patients/year, 2/3 are referred from private obstetricians, the rest from the CHUV). In addition, women with GDM who are followed up by private diabetologists in the canton Vaud (N = around 150 GDM patients/year) will be invited to participate following a similar procedure. Following their first clinical appointment, the study coordinator will explain the study and give the information sheet to the patient. Once the patient has had time to reflect (min. 24 hours) and signed the consent form, she will be included in the study. Partners of women with GDM will also be invited to participate, if the patient agrees that her partner is contacted.

Women without GDM: All pregnant women attending their antenatal appointment policlinique at the CHUV will be invited to participate after their routine screening, once GDM has been excluded. The study coordinator will explain the study and give the study information sheet to the patient. Once the patient has had time to reflect (min. 24 hours) and signed the consent form, she will be included in the study.

Women with GDM participating in *MySweetHeart Trial* will receive for their time, effort as well as the fees for their frequent travels CHF 200 during the prepartum period and CHF 200 during the postpartum period. Partners of women with GDM will receive CHF 50 as compensation for their time and effort. Women without GDM participating in *MySweetHeart Cohort* will receive CHF 100 as compensation for their time and effort.

7.3. Assignment to study groups

A) *MySweetHeart Trial:* Block 1:1 randomisation will be used. A list of blocks of four will be generated in advance and numbered opaque envelopes will be prepared by the data manager. Once the patient has signed the informed consent form, the study coordinator will open the envelopes and communicate the group allocation to the patient (allocation concealment). The sequence of randomisation blocks will be concealed over the course of the study.

B) *MySweetHeart Cohort:* Not applicable.

7.4. Criteria for withdrawal / discontinuation of participants

A) *MySweetHeart Trial:* Participants will be excluded from the study if they wish to withdraw.

B) *MySweetHeart Cohort:* Participants will be excluded from the study if they wish to withdraw

8. STUDY INTERVENTION

8.1. Identity of Investigational Products (treatment / medical device)

Drugs will only be administered for clinical care. No drugs will be administered to the participants within the framework of this study (see 8.2. for description of the multidimensional interdisciplinary intervention and treatment-as-usual condition).

8.1.1. Experimental Intervention (treatment / medical device)

Not applicable.

8.1.2. Control Intervention (standard/routine/comparator treatment / medical device)

Not applicable.

8.1.3. Packaging, Labelling and Supply (re-supply)

Not applicable.

8.1.4. Storage Conditions

Not applicable.

8.2. Administration of experimental and control interventions

This section is applicable only for ***MySweetHeart Trial***

8.2.1. Experimental intervention

The multidimensional interdisciplinary lifestyle and psychosocial intervention will be offered on top of usual care (8.2.2). It will consist of individual sessions (face-to-face or telephone contact) with different members of the interdisciplinary team (dietician, physiotherapist, clinical psychologist or coach) and two group sessions. It will

take place during pregnancy and during the first year postpartum (see Table 1).

The intervention group will have the following key components in addition to the usual care of the control group: Prenatally, 2 physiotherapy and 2 additional dietician consultations, 1 interdisciplinary group session. Based on the patients' context and capacities, individually tailored treatment goals will be established (see Table 4). At study begin, and at 6-8 weeks, patients will be screened for depression. According to a stepped care approach (based on the patient's EPDS score), individual sessions with the clinical psychologist will be offered (see Table 3 for more details). Postnatally, patients will have 4 interdisciplinary individual consultations (dietician, psychologist; physician when needed) focusing on the health of the mother and her offspring, and 1 interdisciplinary group sessions. Throughout the pregnancy and up to 1 year postpartum, patients will be accompanied and supported by a coach (to monitor adherence to the intervention, provide support and teach strategies to work towards the individual goals). During pregnancy, the coach will have 10 min of weekly contact (by phone and/or face to face if patients come for a visit) and tailor the individual intermediate goals to patients' context and capacities in accordance with the respective specialists. During the first year postpartum, the coach will have contact with the mother twice monthly. To increase social support, partners will be invited to attend individual and group sessions during both the prepartum and postpartum period. If partners are unable to attend those sessions, then we will offer a Skype or telephone contact. In addition, small peer support groups will be formed during the interdisciplinary group sessions.

To improve the mental health of participants, the coach will be taught to perform routine screening based on self-report questionnaires and will be trained in cognitive behavioural, mindfulness and motivational strategies, with regular supervision by a clinical psychologist. Participants reporting elevated symptoms (EPDS score of psychopathology on the self-report questionnaires will receive systematic support by the clinical psychologist based on a stepped care approach. Patients who require a psychiatric evaluation including psychotropic medication will be referred to the Psychiatrie de Liaison at the CHUV.

Information about the interplay between mental health, lifestyle behaviour and GDM as well as the link between maternal GDM and the future risks for the mother and the offspring (to improve risk perception) are part of the educational approach. Adherence to the intervention will be enhanced through personal contact based on motivational interviewing and therapeutic patient education.

During the first postpartum year, an educational approach will also be used with the mother (and her partner) to improve their cardio-metabolic and mental health by working on the objectives for their infants (see Table 4).

The follow-up will be provided at the Gestational Diabetes Clinic at the CHUV for all patients that are referred there for clinical care. If the patient is followed by a diabetologist outside of the CHUV, the care that is part of the usual clinical follow-up will be provided by the diabetologist, but the on-top intervention parts and the assessments will be performed at the CHUV. We will ensure close collaboration with the obstetricians, paediatricians, and existing health care networks that form part of the patients' usual clinical care.

Table 3: Intervention features for the mother

DOMAINS	EXPERIMENTAL INTERVENTION GROUP	CONTROL GROUP
DIET	**Free sugars:** Limit the intake to less than 10% of total energy intake (WHO 2015) by avoiding added sugars and sugars naturally present in honey, syrups and fruit juices<br>**Lipids:** Limit the intake to 30% of total energy intake (OFSP 2011) by encouraging healthy food choices, good cooking practices and limiting quantity of added fat<br>**Mindful eating:** Improve eating regulation in developing an awareness of physical hunger and satiety cues	**Free sugars:** Limit the intake of free sugars to less than 10% of total energy intake (WHO 2015) by avoiding added sugars and sugars naturally present in honey, syrups and fruit juices
PHYSICAL ACTIVITY	**Physical activity:** Strongly and specifically encourage 2 x 20 min per day of endurance and resistance activities of moderate intensity:<br>- type: combined endurance and resistance training<br>- frequency: 2 x per day; 7 days per week.<br>- duration: at least 20 min per session.<br>- intensity: moderate intensity (i.e., RPE = 12-14 on Borg's scale).<br>- timing: 1h - 1h30 postprandial.<br>**Sedentary behaviour**: Break sedentary time with physical activity every hour.	**Physical activity:** Provide usual recommendations of 30 min per day of moderate physical activity (Endocrine Society)
MENTAL HEALTH	**Depression: Screening and treatment of moderate depressive symptoms:** Edinburgh Postnatal Depression Scale (EPDS) at first antenatal visit, 6-8 weeks postpartum and 1 year postpartum. Based on the EPDS score, patients will be seen for individual sessions by a clinical psychologist integrated in the team using a stepped care approach (NICE, 2009) with supervision of the Psychiatry Liaison Service. The focus is on prevention and early intervention, with a one-off session offered in case of mild symptoms, and more in case of moderate symptoms (EPDS=10+), or moderate to severe symptoms (EPDS=13+).<br>**Adherence: Assessment and treatment of problems:**<br>In case of problems with adherence interfering with adequate metabolic control, members of the multidisciplinary team may also ask for a clinical assessment by the team clinical psychologist.	**Depression: Screening and referral for moderate to severe depressive symptoms:**<br>Edinburgh Postnatal Depression Scale (EPDS) at first antenatal visit and 1 year postpartum. If EPDS = 13+, referral to Psychiatry Liaison Service.<br>.
SOCIAL SUPPORT	**Healthcare providers:** Ensure that all adequate perinatal support services have been proposed<br>**Peers:** Offer support by group sessions to initiate exchange and contact<br>**Partner:** Integrate the partner in the consultations, groups sessions and the personal established goals	No specific intervention

Table 4: Intervention features for the infant (attained through parent education)

DOMAINS	EXPERIMENTAL INTERVENTION GROUP	CONTROL GROUP
DIET	**Breastfeeding:** encourage for at least 6 months (WHO, 2016)<br>**Soothe:** propose alternative methods than feeding<br>**Hunger and satiety**: recognise cues<br>**Food**: introduce after at least 4 months passed (SSN, SSP 2011)	Provide general recommendation for breastfeeding (WHO, 2016)
SLEEP	Lengthen sleep duration of infant during night	No specific intervention
PHYSICAL ACTIVITY	**Physical activity:** Encourage physical activity during waking hours, reaching 180 min/day at 1 year of age (Tremblay, 2012).<br>**Sedentary behaviour**: No screen time.	No specific intervention
MENTAL HEALTH	**Parental regulation of infant distress and self regulation capacity: I**ncreased through parent education during pre-natal and postnatal workshops and postpartum interdisciplinary sessions and reinforced by coach.	No specific intervention

8.2.2. Control Intervention

Usual clinical follow-up and treatment is based on the current American Diabetes Association [2], the Endocrine Society guidelines [3], and the NICE [111, 112] guidelines.

Patients will be first seen at 24-32 weeks of gestation by a physician and/or a nurse practitioner that follow them up to delivery. During the first visit, patients learn about GDM, how to perform self- control of blood glucose 4x/day (fasting and postprandially) and are encouraged to increase physical activity [113]. They have one appointment with a registered dietician to receive individualized dietary advice to avoid soft drinks and limit added sugar, sweet products and juices. If glucose values remain above targets, twice or more during a 2 week period [113], metformin or insulin treatment is installed depending on glucose values (e.g. insulin for high values), patient preference, and the evaluation of the clinical health care professional. Patients then undergo a 75 g OGTT at 6-8 weeks postpartum and are seen afterwards by the physician or nurse practitioner and a dietician jointly to discuss results and further management. Patients then resume usual care by their health care provider outside of the clinic. A screening for glucose tolerance by fasting glucose and HbA1c is recommended at 1 year postpartum, and then regularly every 1-3 years [2].

This usual clinical follow-up will be provided at the Gestational Diabetes Clinic at the CHUV for all patients that are referred there for clinical care. If the patient is followed by a diabetologist outside of the CHUV, the usual clinical follow-up will be provided by the diabetologist, but the assessments will be done at the CHUV.

Patients with an EPDS score of at least 13 who require a psychiatric evaluation including psychotropic medication will be referred to the Psychiatrie de Liaison at the CHUV.

8.3. Dose / Device modifications

Not applicable.

8.4. Compliance with study intervention

Patients will have regular contact with the coach who will monitor their adherence to the intervention. Brief summaries of the number of clinical appointments and telephone contacts will be kept, as well as the number of missed appointments and their reasons.

8.5. Data Collection and Follow-up for withdrawn participants
Withdrawn participants of the RCT will be contacted by telephone by the coach at 1 year postpartum with the aim of measuring the primary outcomes.

8.6. Trial specific preventive measures
Not applicable.

8.7. Concomitant Interventions (treatments)
All concomitant treatments will be noted in the CRF. If clinically indicated, patients can attend external sessions of psychotherapy during the trial. Participation in structured physical activities such as sports clubs or other physical activity group sessions will be also recorded. Patients can also take any medications, vitamins and micronutrients as clinically indicated.

8.8. Study Drug / Medical Device Accountability
Not applicable.

8.9. Return or Destruction of Study Drug / Medical Device
Not applicable.

9. STUDY ASSESSMENTS

9.1. Study flow chart(s) / table of study procedures and assessments
See Table 1 and 2, and sections 5.1. and 5.2.

9.2. Assessments of outcomes
Written standard operating procedures (SOPs) will be provided for the following outcomes:
- height and weight (mother and infant)
- bioimpedance (mother and infant)
- skinfolds (mother and infant)
- Chester step test
- Jamar dynamometer
- blood sampling (maternal and cord blood)
- fetal ultrasound

9.2.1. Assessment of primary outcome

A) *MySweetHeart Trial:*
Our primary outcomes will be differences between the intervention and control group in (1) the decrease in maternal weight and (2) decrease in maternal symptoms of the Edinburgh Postnatal Depression score at 1 year postpartum, both of which will be assessed by research staff blinded to the group allocation. Maternal weight will be measured by using a calibrated Seca scale. Depression symptom score will be assessed using a validated self-report questionnaire (Edinburgh Postnatal Depression Scale).

B) *MySweetHeart Cohort:*
Primary outcomes will be the surrogate markers of CVD at birth [left ventricular mass index (LVMI) and subclinical atherosclerosis (carotid intima-media thickness; cIMT)].

A neonate echocardiography will be performed 2-4 days after birth (before the mother and the newborn leave the clinic) by two experienced ultrasonographers (Dr N. Sekarski and Dr S. Di Bernado) blinded to the maternal glycemic status and to the fetal echocardiogram. Echocardiography will be performed on a Philips iE33 echocardiogram with a S8-3 or S5- 1MHz transducer, digitally recorded (Xcelera, Philips) and analyzed off-line. Standard echocardiography including M-mode, color and spectral Doppler according to the ASE will be performed [114, 115]. Measurements will be performed according to the standard of the ASE [114, 115]. including: 1) right and left atrial chamber sizes and volumes; 2) LV size, volume and function; 3) LV mass; 4) LV and RV systolic and diastolic function. Z scores based on BSA calculated by the Haycock formula will be used to express these measurements [116-118].

During cardiac echocardiography, carotid ultrasound will be performed 2-7 days after birth (before the mother and the newborn leave the clinic) by two experimented ultrasonographers (Dr N. Sekarski and Dr S. Di Bernardo) blinded to the maternal glycemic status. Carotid IMT measurement will be performed on a Philips iE33 echocardiograph (Philips Medical, Netherland) with a L 11- 5 MHz high-resolution linear array transducer, recorded on a digital system (QLab, Philips Medical Netherlands). Image acquisition will be done according to

the standard of the American Heart Association (AHA) Atherosclerosis, Hypertension and Obesity in Youth Committee of the Council on Cardiovascular Disease in the Young [109]. Image analyses will include: 1) far-wall cIMT; 2) calculation of the mean of maximal cIMT measurements (diameter of carotid artery); 3) calculation of carotid stiffness. Measurements of cIMT will be expressed in mm as mean +/- SD and compared with normal cIMTs [119, 120].

9.2.2. Assessment of secondary outcomes

A) *MySweetHeart Trial:*
Maternal secondary outcomes will be (1) lifestyle behaviours (carbohydrate and fat intake, eating behaviour, breastfeeding, feeding behaviour, physical activity, sleep), aerobic fitness and strength, body composition (total and regional fat mass), cardiometabolic laboratory biomarkers (2) other mental health indicators (anxiety, well-being, perception of social support, parenting stress); during the perinatal and postpartum period (see above under outcomes and in the assessment table for the exact timings).

Offspring secondary outcomes are (1) cardio-metabolic laboratory biomarkers at the time of delivery, body composition (BMI, total fat mass) at birth, 6-8 weeks pp and at 1 yr of age; and (2) mental health indicators (self-regulation, sleep quality and quantity) at 1 yr of age.

More details on assessments are provided in Table 2.

A1) Maternal outcomes:
A1.1) Lifestyle behaviours: Carbohydrate and fat intake will be measured by the Food Frequency Questionnaire, eating behaviour by the French Intuitive Eating Scale, breastfeeding by self-report with a focus on duration and exclusiveness. Physical activity will be measured using an accelerometer that is worn during one week on the wrist (GeneActiv®). Sleep will be measured using the Pittsburgh Sleep Quality Index.

A1.2) Aerobic fitness will be assessed using the Chester step test with VO2max estimation and grip **strength** using a Jamar dynamometer.

A1.3) Body composition measures include bioelectrical impedance analysis using a 4-polar single frequency device (RJL Systems, Model 101A; Detroit, MI, USA), the sum of four four skinfolds (triceps, biceps, subscapular and suprailiac) by triplicate measures using a Harpenden calipers (HSK-BI, British Indicators, UK) as well as Dual-Energy-X-Ray absorptiometry (Lunar®).

A1.4) Cardiometabolic laboratory variables include HDL cholesterol, LDL cholesterol, total cholesterol, triglycerides, HbA1C, insulin, glucose, indices of insulin resistance and insulin secretion, gamma-GT, B12 vitamin, ferritine, free fatty acids, and miRNA. Further, a sample of blood will be kept for future potential analyses. All samples will be managed and stored within the EDM biobank.

A1.5) Additional mental health indicators: Anxiety will be measured with the Anxiety subscale of the Hospital Anxiety and Depression Scale; depression with the Edinburgh Postnatal Depression Scale and Whooley questions. Well-being will be assessed with the WHO Well-Being Index, social support with the Medical Outcomes Study Social Support Survey-short form, and parenting stress with the Parenting Stress Scale-short form.

(2) Offspring outcomes:
A2.1) Cardio-metabolic laboratory biomarkers: A cord blood sample will be collected at the time of delivery. The following variables will be measured: HDL cholesterol, LDL cholesterol, total cholesterol, triglycerides, HbA1C, c-peptide, insulin, creatinine, uric acid, glucose, hs-CRP, gamma-GT, B12 vitamin, and ferritine and miRNA. Further, a sample of blood will be kept for future potential analyses. All samples will be managed and stored within the EDM biobank

A2.2) Body composition measures include height, weight using standardized tools for babies, bioelectrical impedance analysis using a 4-polar single frequency device (RJL Systems, Model 101A; Detroit, MI, USA) and the sum of four four skinfolds (triceps, biceps, subscapular and suprailiac) by triplicate measures using a Harpenden calipers (HSK-BI, British Indicators, UK).

A2.3) Mental health indicators: Self-regulation will be measured with the Difficult Child subscale of the Parenting Stress Index-Short form. Sleep quality and quantity will be measured using the Brief Infant Sleep Questionnaire.

B) *MySweetHeart Cohort:*

Secondary outcomes will be (B.1) the cardiovascular structure and function during the fetal period; (B.2) neonatal adverse cardiovascular risk factors (notably blood cord total cholesterol, glycemia, insulin, c-peptide, hs-CRP).

B.1) A fetal echography will be performed between the 30th and 34th week of gestation by two experimented ultrasonographers (Dr Yvan Mivelaz and Dr Yvan Vial), blinded to the maternal glycemic status, using a Voluson E8 Expert (General Electric Medical Systems, ZIPF, Australia) equipped with a 3–5 MHz convex array sector transducer. A systematic echography will be conducted to detect structural and functional anomalies following international recommendations [121, 122]. Anthropometric measurements and fetal well-being parameters will be measured. Then, a detailed echocardiography of the fetal liver, cardiovascular system and of the materno-fetal circulation will be conducted including bidimensional (2D), M-Mode (M), Doppler (D) and tridimensional (3D) imaging of the following structures: cardiac 4 chambers (2D), RV and LV short axis (2D, M), tricuspid, pulmonary, mitral and aortic valve (2D, D), lateral and medial mitral annulus, and tricuspid annulus (2D, D), umbilical vein, ductus venosus, patent ductus arteriosus, aortic isthmus, umbilical arteries and maternal uterine arteries (2D, D) and liver (2D, 3D). This will allow the assessment of the LV posterior wall and septal thickness, the LV mass[122], the LV and RV systolic and diastolic function, the cardiothoracic ratio, the placental and fetal vascular resistances, the cardiac output and liver volume. Data will be digitally stored to allow off-line analyses. Measures will be expressed as z-scores based on gestational age to allow comparison between fetuses.

B.2) A cord blood sample will be collected at the time of delivery. The following variables will be measured: HDL cholesterol, LDL cholesterol, total cholesterol, triglycerides, HbA1C, c-peptide, insulin, creatinine, uric acid, glucose, hs-CRP, gamma-GT, B12 vitamin, ferritine, free fatty acids, and miRNA. Further, a sample of blood will be kept for future potential analyses. All samples will be managed and stored within the EDM biobank.

9.2.3. Assessment of other outcomes of interest

Not applicable. Note that partners' variables will be body weight and height, eating behaviour and mental health symptoms (anxiety, well-being, perception of social support, parenting stress). These will be assessed in the same manner as described above for the women with GDM.

9.2.4. Assessment of safety outcomes

9.2.4.1. Adverse events

See section 5.4

9.2.4.2. Laboratory parameters

Laboratory results will be monitored and those with clinical relevance will be considered. Should action be required, the information will be transferred to the treating physician.

9.2.4.3. Vital signs

As part of the clinical routine, blood pressure and heart rate will be measured in a sitting position at the beginning and end of the study as well as during the medical pregnancy visits.

9.2.5. Assessments in participants who prematurely stop the study

Participants who are withdrawn from the study prematurely will not be followed up.

9.3. Procedures at each visit

Depending on the group (non GDM; GDM-control; and GDM-intervention), the number and nature of the visits will be different (see Table 1: Overview of visits for the 3 study groups and Table 2: Assessments and events for the 3 study groups). The clinical visits for participants with GDM are detailed in Table 2.

9.3.1. Pre-screening visit

The primary purpose is to recruit pregnant women followed up in the maternity policlinic at the CHUV. As part of the normal pregnancy follow-up at the policlinic, each pregnant woman comes between weeks 22-31 of gestation for GDM screening (fasting glycemia). After this visit, members of the research team will meet them to briefly present the study and give the preliminary information sheet. This first contact with potential participants allows us to evaluate their capacity to understand our explanation and their interest and motivation to take part in the study. Depending on the result of the fasting glycemia, different options will arise:

1) In case of normal fasting glycemia, the research staff will contact them by phone the same day, to ask them if they accept to participate as non-GDM control participants (after having had time to read the preliminary information sheet). If they accept, we send the information/consent letter, and the first visit is planned at the same time as the next normal pregnancy follow-up visit (usually within 2 weeks following the fasting glycemia measurement).

2) In case of high fasting glycemia, the patient will receive a phone call from the policlinic, with an invitation for the oral glucose tolerance testing (routine diagnostic procedure for GDM). In case of normal OGTT, the same recruitment procedure as for the normal fasting glycemia will be applied. In case of abnormal OGTT, the patient

will be transferred to the gestational diabetes consultation at the Maternity Department. Recruitment will be done there along with GDM patients that are referred from obstetricians and diabetologists outside the CHUV.

9.3.2. Recruitment (24-32 weeks of gestation); Visit 1 and Baseline assessment (24-32 weeks of gestation)

(a) Women with GDM:
They will be informed about the study and receive the participant information sheet. When they come back a few days later to attend their first appointment with the dietician, they will report their interest to participate or not in the study. Once they have signed the consent form, baseline assessments will be carried out, including measurements of weight and height, validated self-report questionnaires, tests of aerobic fitness and strength, body composition (skinfolds (callipers), and bioimpedance (see Table of outcome measures and assessments) as well as 3 measures of blood pressure. If the patient agrees, their partners will also be informed about the study and invited to participate using a separate information sheet and consent form. Once he has signed the consent form, research staff will measure his weight and height and ask him to complete validated self-report questionnaires. The medical assistant will draw an additional 31 ml of maternal blood during the planned blood sampling (no extra puncture).

Women recruited by diabetologists outside the CHUV will be informed about the study by the diabetologist, receive the participant information sheet and asked if they agree to participate and be contacted by the study investigator. If they agree to participate, a visit for the baseline assessment will be scheduled.

(b) Women without GDM:
They will be informed about the study and receive the participant information sheet. Once the women have agreed to participate and signed the consent form, the nurse will take an additional 31 ml during the planned blood sampling (no extra puncture). These tubes will be used for the measurement of cardio-metabolic biomarkers. After the pregnancy control visit, the research assistant will take 3 measures of blood pressure, measure height and weight, ask the participant to complete the self-report questionnaires (see Table of outcome measures and assessments), and for her pregravid weight.

9.3.3. Visit 2 (30-34 weeks prenatal)
This visit applies to all participants. During this visit, a fetal utrasound, including cardiac structure and function and liver size will be performed by the obstetricians and fetal cardiologist of the study. The research assistant or nurse will also take 5 measurements of blood pressure.

9.3.5. Visit 3 (during childbirth)
This visit applies to all participants. During delivery, an additional 3 ml will be drawn from the venous line previously installed (no venipuncture) at the entry in the delivery room (normal or elective caesarean surgery). After delivery, blood will be drawn from the cord following clamping.

9.3.6. Visit 4 (2-7 days postpartum)
This visit applies to all participants. Whilst the woman is still hospitalised after delivery, an echocardiography and an cIMT measurement will be performed on the baby. The investigations will be carried out by the pediatric cardiologists of the study. Three maternal blood pressure readings and maternal weight will be taken.

9.3.7. Visit 5 (6-8- weeks postpartum)
This visit does not apply to women without GDM. Women with GDM whilst attending a clinical appointment will be asked to complete a series of validated self-report questionnaires, their height and weight will be measured. They will undergo a bioimpedance assessment and skinfolds measurements (using callipers). 10 ml extra blood will be drawn during the planned clinical blood sampling. Weight, height and skinfolds of their infant will be assessed.

9.3.8. Final visit 6 (1 year postpartum)
This visit does not apply to women without GDM. Women with GDM will be asked to complete validated self-report questionnaires, and will undergo tests of physical fitness and strength, skinfolds, bioimpedance and DEXA (see Table of outcome measures and assessments). 31 ml extra blood will be drawn during the planned clinical blood sampling. Weight, height and skinfolds of their infant will be assessed. For the partners of women with GDM, the research staff will measure their weight and height and ask them to complete validated self-report questionnaires.

10. SAFETY

10.1. Drug studies
Not applicable

10.2. Medical Device Category A studies
Not applicable

11. STATISTICAL METHODS

11.1. Hypothesis

A) *MySweetHeart Trial:*
The primary objectives of the study are to test the effect of a multidimensional lifestyle and psychosocial intervention in women with GDM to improve 1) their metabolic (decrease in maternal weight between 24-32 weeks gestational age and 1 year postpartum at the end of the study) and 2) their mental (decrease in EPDS depression score during the same time period) health. The null hypotheses are that there will be 1) no difference in the reduction of weight and 2) no difference in the reduction of the EPDS depression score between the intervention and the control arm between the study inclusion and the study end.

B) *MySweetHeart Cohort:*
The primary objectives of the study are to assess the effect of GDM on the surrogate markers of cardiovascular disease (CVD) at birth (left ventricular mass index and subclinical atherosclerosis). The null hypotheses are that there are 1) no difference in mean LVMI and 2) not difference in cIMT between children of women with GDM and children of women without GDM.

11.2. Determination of Sample Size

A) *MySweetHeart Trial:*

We have computed the sample size based on the expected difference in primary outcomes (change in maternal metabolic and mental health, i.e., change in weight and in symptoms of the Edinburgh postnatal depression score) between women with GDM allocated to the control and the intervention group. Sample size estimations were performed based on our pilot data.

Regarding maternal weight, we assumed a weight reduction of 2.5 kg [SD: 5] between study inclusion at 24-32 GA, after GDM diagnosis and 1 year postpartum in women allocated to the control group compared to a weight reduction of 5 kg [SD: 6] in women allocated to the intervention group. The required sample size is 78 women in each study group to have a statistical significant difference with a power of 80% and an alpha-level set at 0.05 (two- sided).

This sample size is also sufficient to observe statistical significant differences in the reduction in the Edinburgh Postnatal Depression symptoms score, if we assume that the reduction in depression score between the above mentioned two time points is 2 [SD: 4.3] in women allocated to the control group and 4.0 [SD: 4.4] in women allocated to the intervention group.

Assuming a lost to follow-up of maximum 20%, we will include 100 women in the control and 100 in the intervention group.

B) *MySweetHeart Cohort:*
We have computed the sample size based on the expected difference in primary outcomes (LVMI and cIMT) between children of women with GDM and children of women without GDM. Assuming that LVMI will be 30.0 g/m2.7 [SD: 4.5] in children of mothers without GDM [123] compared to 32.0 g/m2.7 [SD: 4.5] in children of women with GDM, the required sample size is 80 women with GDM and 80 women without GDM to have a statistical significant difference with a power of 80% and an alpha-level set at 0.05 (two-sided).

This sample size is also sufficient to observe statistical significant differences in cIMT at birth if we assume that cIMT at birth is 0.44 mm [SD: 0.04] [108] in newborn of women without GDM and 0.42 mm [SD: 0.04] in newborn of women with GDM. We do not expect to have a substantial lost to follow-up between inclusion and delivery. Nevertheless, in the worst case scenario, we could lose ~20% of the participants.

Therefore, we will include 100 pregnant women with GDM (corresponding to the 100 women of the control group of the trial) and 100 women without GDM.

11.3. Statistical criteria of termination of trial
A) *MySweetHeart Trial:*
Given this is a low risk trial with many important exploratory secondary outcomes, there are no preplanned

criteria to terminate the trial prematurely.
B) *MySweetHeart Cohort:*
Not applicable

11.4. Planned Analyses

11.4.1. Datasets to be analysed, analysis populations
A) *MySweetHeart Trial:*
We will analyze the complete dataset of the intervention and control group of patients with gestational diabetes. In a first step, the population will be analysed as intention to treat. In a second step, we will also perform a per protocol analyses. Subgroup analyses will be performed according to weight status, mental health status (at risk vs not at risk patients), ethnicity, prediabetes status at the intial postpartum evaluation (6-8 weeks pp) as well as sex (for the children).

B) *MySweetHeart Cohort:*
We will analyze the complete dataset of patients with GDM and without GDM.

11.4.2. Primary Analysis

A) *MySweetHeart Trial:*
For the primary analyses, differences in the changes in maternal weight and the EPDS depression symptoms score between inclusion after GDM diagnosis and 1 year postpartum at the end of the study between the 2 groups will be analyzed using linear regression analysis. Analyses will be adjusted for the respective baseline values if there are differences between arms. Variables will be transformed if residuals are not normally distributed. We will include potential confounding variables, if necessary. The potential confounding variables are maternal age, sex of the children, pre-, peri- and early postnatal conditions/complications, and socioeconomic status where applicable. Analyses will be conducted with STATA 14.0.

B) *MySweetHeart Cohort:*
The primary hypothesis is that mean LVMI, respectively cIMT, is larger in children of women with GDM than in children of women without GDM. This hypothesis will be tested using linear regression analyses, GDM being the exposure, LVMI (respectively cIMT) being the outcome, and with adjustment for potential confounding factors (i.e., maternal obesity, weight gain during pregnancy, smoking, socio-economic status, or family history of cardio-metabolic diseases). Regression coefficient will be reported with 95% confidence interval. Analyses will be conducted with STATA 14.0.

11.4.3. Secondary Analyses
A) *MySweetHeart Trial:*

For secondary outcomes, the same type of statistical analyses will be conducted as for the primary outcomes. The analyses will be performed for differences in changes between groups and differences between groups at different time points (baseline at inclusion, delivery, 6-8 weeks and 1 year postpartum) in maternal metabolic health outcomes, maternal mental health outcomes and offspring metabolic and mental health outcomes that will be tested using linear regression analysis. Associations between outcomes will also be tested using linear regression analyses.

We will also compare the proportion of patients meeting guidelines for gestational weight gain and weight retention at 1 year postpartum between the two arms using logistic regression analyses and evaluate, how glucose and insulin and other adipokine concentrations at baseline and at 6-8 weeks postpartum can predict glucose intolerance at 1 year postpartum. For the partners, we will evaluate changes between groups and differences between groups at different time points (baseline at inclusion, 1 year postpartum) in weight and paternal eating behavior and mental health outcomes.

We will include potential covariates and test moderators of interest. **Covariates:** Analyses will be adjusted for maternal age, sex (for the children), baseline parameters, if applicable. Analyses will also be adjusted for BMI, EPDS depression score, pre-, peri- and early postnatal conditions/complications, socioeconomic status where applicable. **Moderators:** Where applicable, weight status and ethnicity as well as prediabetes status at the intial postpartum evaluation (6-8 weeks pp) and partners presence will be tested as possible moderators. **Subgroup analyses** will be performed according to weight status, mental health status (at risk vs not at risk patients), ethnicity, prediabetes status at the intial postpartum evaluation (6-8 weeks pp).

A process evaluation will also be performed (number of individual and workshop visits and coach contact, presence of partner).

Furthermore, post-hoc exploratory analyses will be conducted but described as such in publications.

B) *MySweetHeart Cohort:*
For secondary outcomes, the same type of statistical analyses will be conducted as for the primary outcomes. We also plan to conduct mediation analyses to estimate the direct effect of GDM on LVMI (respectively cIMT). Our hypothesis is that there is a direct effect of GDM on offspring's LVMI (respectively cIMT) and an indirect effect through the mediator large for gestational age (LGA) [95]. A series of linear regression analyses with and without adjustment for birth weight, accounting for potential confounding factors, will allow estimates these direct and indirect effects [124, 125]. Furthermore, post-hoc exploratory analyses will be conducted but described as such in publications.

11.4.4. Interim analyses
A) *MySweetHeart Trial:*
In this low risk and relatively small and relatively short trial, there are no plans to perform an interim analysis
B) *MySweetHeart Cohort:*
Not applicable.

11.4.5. Safety analysis
A) *MySweetHeart Trial:*
At the end of the study, the proportion of adverse events such as strains and fractures as well as the proportion of patients with prematurity will be analysed by logistic regression analyses.

B) *MySweetHeart Cohort:*
Not applicable.

11.4.6. Deviation(s) from the original statistical plan
Additional exploratory analyses will be performed, also according to the new current scientific knowledge (the study will be finished in 4-5 years).

11.5. Handling of missing data and drop-outs
For some analyses, missing values are dealt with using either multiple imputation or maximum likelihood procedures which have repeatedly shown to provide unbiased parameter estimates under the missing at random (MAR) condition.

12. QUALITY ASSURANCE AND CONTROL
All clinicians delivering the intervention will receive appropriate training and will be supervised on a weekly basis. The content and frequency of all face-to-face or telephone contacts with the participants will be recorded.
QA and QC systems will be maintained using written SOPs and working instructions that will be delivered to all staff involved in the study, depending on their duties.

12.1. Data handling and record keeping / archiving
Paper Case Report Forms will be kept in a locked filing cabinet. Only authorised persons of the research team will have access to these as well as the secure electronic database (Secutrial).

12.1.1. Case Report Forms
Study data will be recorded on paper Case Report Forms (CRF). For each study participant a coded ID number is attributed and used on the CRF. Each CRF will be kept up-to-date along the different visits. The research team will be authorised to make entries in the CRF. Initials of the person entering data on the CRF will allow identifying who made the entries. The data will then be entered into a secure electronic database (Secutrial) using double data entry for the primary outcomes. The Secutrial system is implemented and secured within the CHUV.

12.1.2. Specification of source documents
Source documents are signed informed consent forms, randomisation number, concomitant medication, visit dates, results of relevant examinations (Bioimpedance, DEXA, anthropometric measurements and echography), and CRF. All documents will be kept in a locked filing cabinet at the Department of Endocrinology, Diabetes and Metabolism, in the Department of Pediatric Cardiology for the control non DGM participants, or on the secure servers of CHUV for electronic recordings

12.1.3. Record keeping / archiving
All study data will be archived for a minimum of 10 years after study termination or premature termination of the clinical trial. Paper documents will be stored at the Department of Endocrinology, Diabetes and Metabolism, and in the Department of Pediatric Cardiology. All electronic data will be securely stored in Secutrial and will be only accessible to the PIs and Co PI's.

12.2. Data management
All study data will be entered by research staff (Ph.D. students and study co-ordinators). All data will be pre-coded and stored in a secured database (Secutrial), which will be regularly updated by the CHUV IT Service. Double data entry will be done for the primary outcomes. For the rest of the data, a random 5% will be double-checked.

12.2.1. Data Management System
We will use Secutrial, for which the PIs will carry responsibility. We are already using this system in the GDM Service after having tested and piloted it. A new database will be created for the purpose of this study.

12.2.2. Data security, access and back-up
Only the PIs and Co-PIs will have access to the secure database, which is automatically backed up by the CHUV IT Service.

12.2.3. Analysis and archiving
For analysis by the trial statistician, data will be extracted and imported into a STATA database. All study data will be archived for a minimum of 10 years after study termination or premature termination of the clinical trial. Paper documents will be stored at the Department of Endocrinology, Diabetes and Metabolism, and in the Department of Pediatric Cardiology

12.2.4. Electronic and central data validation
Not applicable.

12.3. Monitoring
Monitoring will be organized by the PI's and performed as follows:

Initial visit (before the start of recruitment)
- check that all members of the clinical and research team understand their roles and have received the appropriate training
- check that all study procedures (as documented by SOPs) are put into place
- check that all study documents (CRF) are prepared

Intermediate visit (after recruitment of 20 patients (10% of study population))
- check that all study procedures are followed according to SOPs and protocol
- check the study documents (CRF, signed consent form) and their storage in a safe place
- check the randomisation procedure and participant confidentiality

The source data/documents will be accessible to monitors and questions are answered during monitoring.

12.4 Audits and Inspections
All study documentation and the source data/documents will be accessible to auditors/inspectors of the CEC and questions will be answered during inspections. All involved parties will ensure that the participant's data are kept strictly confidential.

12.5 Confidentiality, Data Protection
Direct access to source documents will be permitted for purposes of audits and inspections (12.4) Only the PI's and Co-PI's will have access to protocol, dataset, statistical code, etc during and after the study (publication, dissemination).

12.6. Storage of biological material and related health data
Blood samples will be collected from the participants and their offspring. The informed consent they will sign contains a specific paragraph, detailed in the information letter, explaining the kind and purpose of storage of biological samples.

The coded blood samples will be stored for 10 years in a biobank, named *MySweetHeart Biobank*, which will be held and maintained within the biobank of the Department of Endocrinology, Diabetes and Metabolism. This biobank has been registered by the CER-VD, and the BIL. The management of MySweetHeart biobank will belong to PI's of the study, who will respect standard regulation and requirements for biobank management. The biobank management software SLIMS, used by the EDM biobank management, will be developed specifically and used for this study.

All health data related to the biobank will be held in the Secutrial database. The coding file allowing the identification of the participants, stays under the responsibility of the study PI's, and will not be available to anyone except for inspection purposes if required.

13. PUBLICATION AND DISSEMINATION POLICY

See study collaboration agreement

14. FUNDING AND SUPPORT

14.1. Funding

A) *MySweetHeart Trial:* Unrestricted educational grants from Gottfried und Julia Bangerter-Rhyner-Stiftung, Dreyfus Foundation, and Swiss Diabetes Foundation. After having obtained some pilot data, a request will be submitted to the Swiss National Foundation to obtain additional funding.

B) *MySweetHeart Cohort:* Swiss National Foundation (SNF; grant 32003_B 163240/1).

14.2. Other Support

Not applicable

15. INSURANCE

This study is categorized as « Other clinical study » according to art. 61 OClin Category A. Therefore, no insurance is required.

16. REFERENCES

1. Metzger, B.E., et al., *Hyperglycemia and adverse pregnancy outcomes.* The New England Journal Of Medicine, 2008. **358**(19): p. 1991-2002.
2. Association, A.D., *Standards of Medical Care in Diabetes—2016: Summary of Revisions.* Diabetes Care, 2016. **39**(Supplement 1): p. S4-S5.
3. Blumer, I., et al., *Diabetes and pregnancy: an endocrine society clinical practice guideline.* J Clin Endocrinol Metab, 2013. **98**(11): p. 4227-49.
4. Ryser Rüetschi, J., et al., *Fasting glycaemia to simplify screening for gestational diabetes.* BJOG: An International Journal of Obstetrics & Gynaecology, 2016.
5. Bellamy, L., et al., *Type 2 diabetes mellitus after gestational diabetes: a systematic review and meta-analysis.* Lancet, 2009. **373**(9677): p. 1773-9.
6. Lauenborg, J., et al., *Increasing Incidence of Diabetes After Gestational Diabetes A long-term follow-up in a Danish population.* Diabetes Care, 2004. **27**(5): p. 1194-1199.
7. Retnakaran, R. and B.R. Shah, *Mild glucose intolerance in pregnancy and risk of cardiovascular disease: a population-based cohort study.* Canadian Medical Association Journal, 2009. **181**(6-7): p. 371-376.
8. Harreiter, J., G. Dovjak, and A. Kautzky-Willer, *Gestational diabetes mellitus and cardiovascular risk after pregnancy.* Women's Health, 2014. **10**(1): p. 91-108.
9. Nicklas, J., et al., *Factors Associated with Depressive Symptoms in the Early Postpartum Period Among Women with Recent Gestational Diabetes Mellitus.* Maternal and Child Health Journal, 2013. **17**(9): p. 1665-1672.
10. Cosson, E., et al., *Psychosocial deprivation in women with gestational diabetes mellitus is associated with poor fetomaternal prognoses: an observational study.* BMJ Open, 2015. **5**(3).
11. Yee, L.M., et al., *Factors promoting diabetes self-care among low-income, minority pregnant women.* J Perinatol, 2016. **36**(1): p. 13-18.
12. Koh, D., et al., *Health-enhancing physical activity behaviour and related factors in postpartum women with recent gestational diabetes mellitus.* Journal of Science and Medicine in Sport, 2010. **13**(1): p. 42-45.
13. Kim, C., et al., *Self-Efficacy, Social Support, and Associations With Physical Activity and Body Mass Index Among Women With Histories of Gestational Diabetes Mellitus.* The Diabetes Educator, 2008. **34**(4): p. 719-728.
14. Morrison, M., et al., *Factors associated with early cessation of breastfeeding in women with gestational diabetes mellitus.* Women and Birth, 2015.
15. Räisänen, S., et al., *Risk factors for and perinatal outcomes of major depression during pregnancy: a population-based analysis during 2002–2010 in Finland.* BMJ Open, 2014. **4**(11).
16. Poston, L., *Maternal obesity, gestational weight gain and diet as determinants of offspring long term health.* Best Practice & Research Clinical Endocrinology & Metabolism, 2012. **26**(5): p. 627-639.
17. Hanson, M. and P. Gluckman, *Early developmental conditioning of later health and disease: physiology or pathophysiology?* Physiological reviews, 2014. **94**(4): p. 1027-1076.
18. Simeoni, U., et al., *Epigenetics and neonatal nutrition.* Early human development, 2014. **90**: p. S23-S24.
19. Gluckman, P.D., et al., *Effect of in utero and early-life conditions on adult health and disease.* New England Journal of Medicine, 2008. **359**(1): p. 61-73.
20. Nehring, I., et al., *Gestational diabetes predicts the risk of childhood overweight and abdominal circumference independent of maternal obesity.* Diabetic Medicine, 2013. **30**(12): p. 1449-1456.
21. Crume, T., et al., *Association of exposure to diabetes in utero with adiposity and fat distribution in a multiethnic population of youth: the Exploring Perinatal Outcomes among Children (EPOCH) Study.* Diabetologia, 2011. **54**(1): p. 87-92.
22. Chandler-Laney, P.C., et al., *Overweight status and intrauterine exposure to gestational diabetes are associated with children's metabolic health.* Pediatric obesity, 2012. **7**(1): p. 44-52.
23. Mehta, S.H., M. Kruger, and R.J. Sokol, *Is maternal diabetes a risk factor for childhood obesity?* Journal of Maternal-Fetal and Neonatal Medicine, 2012. **25**(1): p. 41-44.
24. Pettitt, D.J., et al., *Maternal glucose at 28 weeks of gestation is not associated with obesity in 2-year-old offspring: the Belfast Hyperglycemia and Adverse Pregnancy Outcome (HAPO) family study.* Diabetes care, 2010. **33**(6): p. 1219-23.
25. Clausen, T.D., et al., *High prevalence of type 2 diabetes and pre-diabetes in adult offspring of women*

with gestational diabetes mellitus or type 1 diabetes the role of intrauterine hyperglycemia. Diabetes care, 2008. **31**(2): p. 340-346.

26. Mathers, J.C., G. Strathdee, and C.L. Relton, *Induction of epigenetic alterations by dietary and other environmental factors.* Adv Genet, 2010. **71**: p. 3-39.
27. Su, L.J., et al., *Epigenetic contributions to the relationship between cancer and dietary intake of nutrients, bioactive food components, and environmental toxicants.* Frontiers in genetics, 2011. **2**.
28. Chen, X., et al., *Characterization of microRNAs in serum: a novel class of biomarkers for diagnosis of cancer and other diseases.* Cell research, 2008. **18**(10): p. 997-1006.
29. Hunter, M.P., et al., *Detection of microRNA expression in human peripheral blood microvesicles.* PloS one, 2008. **3**(11): p. e3694.
30. Fernandez-Twinn, D.S., et al., *Downregulation of IRS-1 in adipose tissue of offspring of obese mice is programmed cell-autonomously through post-transcriptional mechanisms.* Molecular metabolism, 2014. **3**(3): p. 325-333.
31. Nicholas, L.M., et al., *Differential effects of maternal obesity and weight loss in the periconceptional period on the epigenetic regulation of hepatic insulin-signaling pathways in the offspring.* The FASEB Journal, 2013. **27**(9): p. 3786-3796.
32. Russo, M.D., et al., *Gestational weight gain and adiposity, fat distribution, metabolic profile, and blood pressure in offspring: the IDEFICS project.* International Journal of Obesity, 2013. **37**(7): p. 914-919.
33. Black, M.H., et al., *The relative contribution of prepregnancy overweight and obesity, gestational weight gain, and IADPSG-defined gestational diabetes mellitus to fetal overgrowth.* Diabetes Care, 2013. **36**(1): p. 56-62.
34. Reynolds, D., E. Hennessy, and E. Polek, *Is breastfeeding in infancy predictive of child mental well-being and protective against obesity at 9 years of age?* Child Care Health Dev, 2013.
35. Harrod, C.S., et al., *Physical Activity in Pregnancy and Neonatal Body Composition: The Healthy Start Study.* Obstetrics & Gynecology, 2014. **124**(2, PART 1): p. 257-264.
36. Dasgupta, K., et al., *Gestational Diabetes Mellitus in Mothers as a Diabetes Predictor in Fathers: A Retrospective Cohort Analysis.* Diabetes care, 2015. **38**(9): p. e130-e131.
37. Ng, M., et al., *Global, regional, and national prevalence of overweight and obesity in children and adults during 1980–2013: a systematic analysis for the Global Burden of Disease Study 2013.* The Lancet, 2014.
38. Dode, M.A.S.d.O. and I.S. dos Santos, *Non classical risk factors for gestational diabetes mellitus: a systematic review of the literature.* Cadernos De Saúde Pública, 2009. **25 Suppl 3**: p. S341-S359.
39. Martins, C., L. Morgan, and H. Truby, *A review of the effects of exercise on appetite regulation: an obesity perspective.* International journal of obesity (2005), 2008. **32**(9): p. 1337-1347.
40. Bowers, K., et al., *A prospective study of prepregnancy dietary fat intake and risk of gestational diabetes.* Am J Clin Nutr, 2012. **95**(2): p. 446-53.
41. Bao, W., et al., *Prepregnancy Dietary Protein Intake, Major Dietary Protein Sources, and the Risk of Gestational Diabetes Mellitus A prospective cohort study.* Diabetes care, 2013: p. DC_122018.
42. Hosler, A.S., S.G. Nayak, and A.M. Radigan, *Stressful events, smoking exposure and other maternal risk factors associated with gestational diabetes mellitus.* Paediatr Perinat Epidemiol, 2011. **25**(6): p. 566-74.
43. Spirito, A., et al., *Stress, coping, and social support as mediators of the emotional status of women with gestational diabetes.* Psychology & Health, 1991. **5**(2): p. 111-120.
44. Horsch, A., et al., *Stress exposure and physiological stress responses are related to glucose concentrations during pregnancy.* British Journal of Health Psychology, Accepted for publication.
45. Giesbrecht, G.F., et al., *Psychological distress and salivary cortisol covary within persons during pregnancy.* Psychoneuroendocrinology, 2012. **37**(2): p. 270-279.
46. Leppänen, M., et al., *Physical Activity During Pregnancy: Predictors of Change, Perceived Support and Barriers Among Women at Increased Risk of Gestational Diabetes.* Maternal and Child Health Journal, 2014. **18**(9): p. 2158-2166.
47. Lydon, K., et al., *Psychological stress associated with diabetes during pregnancy: a pilot study.* Ir Med J, 2012. **105**(Suppl 5): p. 26-28.
48. Ferrara, A., et al., *A pregnancy and postpartum lifestyle intervention in women with gestational diabetes mellitus reduces diabetes risk factors: a feasibility randomized control trial.* Diabetes care, 2011. **34**(7): p. 1519-25.
49. Ferrara, A., et al., *A pragmatic cluster randomized clinical trial of diabetes prevention strategies for*

women with gestational diabetes: design and rationale of the Gestational Diabetes' Effects on Moms (GEM) study. BMC Pregnancy and Childbirth, 2014. **14**(1): p. 21.

50. Moreno-Castilla, C., et al., *Low-Carbohydrate Diet for the Treatment of Gestational Diabetes Mellitus A randomized controlled trial.* Diabetes care, 2013. **36**(8): p. 2233-2238.
51. Louie, J.C.Y., et al., *Glycemic index and pregnancy: a systematic literature review.* Journal of nutrition and metabolism, 2011. **2010**.
52. Han, S., et al., *Different types of dietary advice for women with gestational diabetes mellitus.* Cochrane Database Syst Rev, 2013. **3**: p. CD009275.
53. Risérus, U., W.C. Willett, and F.B. Hu, *Dietary fats and prevention of type 2 diabetes.* Progress in lipid research, 2009. **48**(1): p. 44-51.
54. Schoenaker, D.A.J.M., et al., *The Role of Energy, Nutrients, Foods, and Dietary Patterns in the Development of Gestational Diabetes Mellitus: A Systematic Review of Observational Studies.* Diabetes Care, 2016. **39**(1): p. 16-23.
55. Bao, W., et al., *Low Carbohydrate–Diet Scores and Long-term Risk of Type 2 Diabetes Among Women With a History of Gestational Diabetes Mellitus: A Prospective Cohort Study.* Diabetes Care, 2016. **39**(1): p. 43-49.
56. Hernandez, T.L., et al., *Women With Gestational Diabetes Mellitus Randomized to a Higher–Complex Carbohydrate/Low-Fat Diet Manifest Lower Adipose Tissue Insulin Resistance, Inflammation, Glucose, and Free Fatty Acids: A Pilot Study.* Diabetes Care, 2016. **39**(1): p. 39-42.
57. American Diabetes, A., *Standards of medical care in diabetes--2014.* Diabetes care, 2014. **37 Suppl 1**: p. S14-80.
58. Melzer, K., et al., *Physical activity and pregnancy.* Sports Medicine, 2010. **40**(6): p. 493-507.
59. Ruchat, S.M. and M.F. Mottola, *The important role of physical activity in the prevention and management of gestational diabetes mellitus.* Diabetes/metabolism research and reviews, 2013. **29**(5): p. 334-346.
60. May, L.E., et al., *Aerobic exercise during pregnancy influences infant heart rate variability at one month of age.* Early human development, 2014. **90**(1): p. 33-38.
61. Youngwanichsetha, S., S. Phumdoung, and T. Ingkathawornwong, *The effects of mindfulness eating and yoga exercise on blood sugar levels of pregnant women with gestational diabetes mellitus.* Applied Nursing Research, (0).
62. Brown, H.L., *ACOG Guidelines at a Glance: Gestational Diabetes Mellitus.* Obstet Gynecol, 2013. **122**: p. 406-16.
63. Gabbe, S.G., et al., *Promoting health after gestational diabetes: a National Diabetes Education Program call to action.* Obstetrics and gynecology, 2012. **119**(1): p. 171.
64. Tovar, A., et al., *Peer Reviewed: Postpartum Screening for Diabetes Among Women With a History of Gestational Diabetes Mellitus.* Preventing chronic disease, 2011. **8**(6).
65. Chasan-Taber, L., *Lifestyle Interventions to Reduce Risk of Diabetes among Women with Prior Gestational Diabetes Mellitus.* Best Practice & Research Clinical Obstetrics & Gynaecology, 2014.
66. Sattar, N., S. Wannamethee, and N. Forouhi, *Novel biochemical risk factors for type 2 diabetes: pathogenic insights or prediction possibilities?* Diabetologia, 2008. **51**(6): p. 926-940.
67. Guay, C. and R. Regazzi, *Circulating microRNAs as novel biomarkers for diabetes mellitus.* Nature Reviews Endocrinology, 2013.
68. Wang, T.J., et al., *Metabolite profiles and the risk of developing diabetes.* Nature medicine, 2011. **17**(4): p. 448-453.
69. Wang-Sattler, R., et al., *Novel biomarkers for pre-diabetes identified by metabolomics.* Molecular systems biology, 2012. **8**(1).
70. Paul, I.M., et al., *Opportunities for the primary prevention of obesity during infancy.* Advances in pediatrics, 2009. **56**(1): p. 107.
71. Crume, T.L., et al., *Long-term impact of neonatal breastfeeding on childhood adiposity and fat distribution among children exposed to diabetes in utero.* Diabetes care, 2011. **34**(3): p. 641-5.
72. Stifter, C.A., et al., *Parent use of food to soothe infant/toddler distress and child weight status. An exploratory study.* Appetite, 2011. **57**(3): p. 693-699.
73. Wardle, J., et al., *Parental feeding style and the inter-generational transmission of obesity risk.* Obesity research, 2002. **10**(6): p. 453-462.
74. Paul, I., et al., *The Intervention Nurses Start Infants Growing on Healthy Trajectories (INSIGHT) study.* BMC Pediatrics, 2014. **14**(1): p. 184.

75. Birch, L.L., S. Anzman-Frasca, and I.M. Paul, *Starting early: Obesity prevention during infancy.* 2012.

76. Seshiah, V. and V. Balaji, *Primordial prevention: maternal health and diabetes.* Diabetes Management, 2013. **3**(4): p. 333-341.

77. Kussmann, M., et al., *Perspective: a systems approach to diabetes research.* Frontiers in genetics, 2013. **4**.

78. Ullmo, S., et al., *Pathologic ventricular hypertrophy in the offspring of diabetic mothers: a retrospective study.* European heart journal, 2007. **28**(11): p. 1319-25.

79. Veille, J.C., et al., *Fetal cardiac size in normal, intrauterine growth retarded, and diabetic pregnancies.* American journal of perinatology, 1993. **10**(4): p. 275-9.

80. Nolan, C.J., P. Damm, and M. Prentki, *Type 2 diabetes across generations: from pathophysiology to prevention and management.* Lancet, 2011. **378**(9786): p. 169-81.

81. Hanson, M.A. and P.D. Gluckman, *Early developmental conditioning of later health and disease: physiology or pathophysiology?* Physiol Rev, 2014. **94**(4): p. 1027-76.

82. Simeoni, U., et al., *Epigenetics and neonatal nutrition.* Early Hum Dev, 2014. **90 Suppl 2**: p. S23-4.

83. Gluckman, P.D., et al., *Effect of in utero and early-life conditions on adult health and disease.* The New England journal of medicine, 2008. **359**(1): p. 61-73.

84. Hivert, M.F., et al., *Metabolomics in the developmental origins of obesity and its cardiometabolic consequences.* J Dev Orig Health Dis, 2015: p. 1-14.

85. Clausen, T.D., et al., *Overweight and the metabolic syndrome in adult offspring of women with diet-treated gestational diabetes mellitus or type 1 diabetes.* The Journal of clinical endocrinology and metabolism, 2009. **94**(7): p. 2464-70.

86. Pettitt, D.J., et al., *Excessive obesity in offspring of Pima Indian women with diabetes during pregnancy.* The New England journal of medicine, 1983. **308**(5): p. 242-5.

87. Moore, T.R., *Fetal exposure to gestational diabetes contributes to subsequent adult metabolic syndrome.* American journal of obstetrics and gynecology, 2010. **202**(6): p. 643-9.

88. Lawlor, D.A., P. Lichtenstein, and N. Langstrom, *Association of maternal diabetes mellitus in pregnancy with offspring adiposity into early adulthood: sibling study in a prospective cohort of 280,866 men from 248,293 families.* Circulation, 2011. **123**(3): p. 258-65.

89. Clausen, T.D., et al., *High prevalence of type 2 diabetes and pre-diabetes in adult offspring of women with gestational diabetes mellitus or type 1 diabetes: the role of intrauterine hyperglycemia.* Diabetes care, 2008. **31**(2): p. 340-6.

90. Parretti, E., et al., *Sonographic evaluation of fetal growth and body composition in women with different degrees of normal glucose metabolism.* Diabetes care, 2003. **26**(10): p. 2741-8.

91. Lawlor, D.A., et al., *Association of existing diabetes, gestational diabetes and glycosuria in pregnancy with macrosomia and offspring body mass index, waist and fat mass in later childhood: findings from a prospective pregnancy cohort.* Diabetologia, 2010. **53**(1): p. 89-97.

92. Lawlor, D.A., et al., *Does maternal weight gain in pregnancy have long-term effects on offspring adiposity? A sibling study in a prospective cohort of 146,894 men from 136,050 families.* The American journal of clinical nutrition, 2011. **94**(1): p. 142-8.

93. Fetita, L.S., et al., *Consequences of fetal exposure to maternal diabetes in offspring.* The Journal of clinical endocrinology and metabolism, 2006. **91**(10): p. 3718-24.

94. Burguet, A., *Long-term outcome in children of mothers with gestational diabetes.* Diabetes & metabolism, 2010. **36**(6 Pt 2): p. 682-94.

95. Vohr, B.R. and C.M. Boney, *Gestational diabetes: the forerunner for the development of maternal and childhood obesity and metabolic syndrome?* The journal of maternal-fetal & neonatal medicine : the official journal of the European Association of Perinatal Medicine, the Federation of Asia and Oceania Perinatal Societies, the International Society of Perinatal Obstetricians, 2008. **21**(3): p. 149-57.

96. Reece, E.A., G. Leguizamon, and A. Wiznitzer, *Gestational diabetes: the need for a common ground.* Lancet, 2009. **373**(9677): p. 1789-97.

97. Mitanchez, D., A. Burguet, and U. Simeoni, *Infants born to mothers with gestational diabetes mellitus: mild neonatal effects, a long-term threat to global health.* J Pediatr, 2014. **164**(3): p. 445-50.

98. Chandler-Laney, P.C., et al., *Maternal glucose concentration during pregnancy predicts fat and lean mass of prepubertal offspring.* Diabetes care, 2011. **34**(3): p. 741-5.

99. Kiserud, T., *Diabetes in pregnancy: scanning the wrong horizon?* Ultrasound in obstetrics & gynecology : the official journal of the International Society of Ultrasound in Obstetrics and Gynecology, 2010. **36**(3): p. 266-7.

100. Kessler, J., et al., *Venous liver blood flow and regulation of human fetal growth: evidence from macrosomic fetuses.* American journal of obstetrics and gynecology, 2011. **204**(5): p. 429 e1-7.

101. Ebbing, C., S. Rasmussen, and T. Kiserud, *Fetal hemodynamic development of macrosomic growth.* Ultrasound in obstetrics & gynecology : the official journal of the International Society of Ultrasound in Obstetrics and Gynecology, 2011.

102. Tchirikov, M., et al., *Liver blood perfusion as a possible instrument for fetal growth regulation.* Placenta, 2002. **23 Suppl A**: p. S153-8.

103. Mirghani, H., et al., *Gestational diabetes mellitus: fetal liver length measurements between 21and 24 weeks' gestation.* Journal of clinical ultrasound : JCU, 2007. **35**(1): p. 34-7.

104. Anderson, N.G., et al., *Reproducibility of sonographic assessment of fetal liver length in diabetic pregnancies.* Ultrasound in obstetrics & gynecology : the official journal of the International Society of Ultrasound in Obstetrics and Gynecology, 2008. **31**(5): p. 529-34.

105. Rijpert, M., et al., *Cardiac function in 7-8-year-old offspring of women with type 1 diabetes.* Exp Diabetes Res, 2011. **2011**: p. 564316.

106. Nolan, C.J., *Controversies in gestational diabetes.* Best practice & research. Clinical obstetrics & gynaecology, 2011. **25**(1): p. 37-49.

107. Landon, M.B., *Is there a benefit to the treatment of mild gestational diabetes mellitus?* American journal of obstetrics and gynecology, 2010. **202**(6): p. 649-53.

108. Sorof, J.M., et al., *Carotid artery intimal-medial thickness and left ventricular hypertrophy in children with elevated blood pressure.* Pediatrics, 2003. **111**(1): p. 61-6.

109. Urbina, E.M., et al., *Noninvasive assessment of subclinical atherosclerosis in children and adolescents: recommendations for standard assessment for clinical research: a scientific statement from the American Heart Association.* Hypertension, 2009. **54**(5): p. 919-50.

110. McGill, H.C., Jr., C.A. McMahan, and S.S. Gidding, *Preventing heart disease in the 21st century: implications of the Pathobiological Determinants of Atherosclerosis in Youth (PDAY) study.* Circulation, 2008. **117**(9): p. 1216-27.

111. Health, N.C.C.f.M., *Antenatal and postnatal mental health: the NICE guideline on clinical management and service guidance.* 2014.

112. Health, N.C.C.f.M. *Depression: the treatment and management of depression in adults (updated edition).* 2010. British Psychological Society.

113. Penn, L., et al., *Importance of weight loss maintenance and risk prediction in the prevention of type 2 diabetes: analysis of European Diabetes Prevention Study RCT.* PloS one, 2013. **8**(2): p. e57143.

114. Lai, W.W., et al., *Guidelines and standards for performance of a pediatric echocardiogram: a report from the Task Force of the Pediatric Council of the American Society of Echocardiography.* Journal of the American Society of Echocardiography : official publication of the American Society of Echocardiography, 2006. **19**(12): p. 1413-30.

115. Lopez, L., et al., *Recommendations for quantification methods during the performance of a pediatric echocardiogram: a report from the Pediatric Measurements Writing Group of the American Society of Echocardiography Pediatric and Congenital Heart Disease Council.* Journal of the American Society of Echocardiography : official publication of the American Society of Echocardiography, 2010. **23**(5): p. 465-95; quiz 576-7.

116. Daubeney, P.E., et al., *Relationship of the dimension of cardiac structures to body size: an echocardiographic study in normal infants and children.* Cardiology in the young, 1999. **9**(4): p. 402-10.

117. Pettersen, M.D., et al., *Regression equations for calculation of z scores of cardiac structures in a large cohort of healthy infants, children, and adolescents: an echocardiographic study.* Journal of the American Society of Echocardiography : official publication of the American Society of Echocardiography, 2008. **21**(8): p. 922-34.

118. Haycock, G.B., G.J. Schwartz, and D.H. Wisotsky, *Geometric method for measuring body surface area: a height-weight formula validated in infants, children, and adults.* The Journal of pediatrics, 1978. **93**(1): p. 62-6.

119. Sass, C., et al., *Intima-media thickness and diameter of carotid and femoral arteries in children, adolescents and adults from the Stanislas cohort: effect of age, sex, anthropometry and blood pressure.* Journal of hypertension, 1998. **16**(11): p. 1593-602.

120. Jourdan, C., et al., *Normative values for intima-media thickness and distensibility of large arteries in healthy adolescents.* Journal of hypertension, 2005. **23**(9): p. 1707-15.

121. Lee, W., et al., *ISUOG consensus statement: what constitutes a fetal echocardiogram?* Ultrasound in obstetrics & gynecology : the official journal of the International Society of Ultrasound in Obstetrics and

Gynecology, 2008. **32**(2): p. 239-42.

122. Reichek, N., et al., *Anatomic validation of left ventricular mass estimates from clinical two-dimensional echocardiography: initial results.* Circulation, 1983. **67**(2): p. 348-52.

123. Maggio, A.B., et al., *Associations among obesity, blood pressure, and left ventricular mass.* The Journal of pediatrics, 2008. **152**(4): p. 489-93.

124. VanderWeele, T.J., S.L. Mumford, and E.F. Schisterman, *Conditioning on intermediates in perinatal epidemiology.* Epidemiology, 2012. **23**(1): p. 1-9.

125. Chiolero, A., J.S. Kaufman, and G. Paradis, *Why adjustment for current weight can bias the estimate of the effect of birth weight on blood pressure: shedding light using causal graphs.* Journal of hypertension, 2012. **30**(5): p. 1042-5.
